# Generation of Neutralizing Antibodies and Divergence of SIVmac239 in Cynomolgus Macaques Following Short-Term Early Antiretroviral Therapy

**DOI:** 10.1371/journal.ppat.1001084

**Published:** 2010-09-02

**Authors:** Gülşen Özkaya Şahin, Emma J. Bowles, Joe Parker, Hannes Uchtenhagen, Enas Sheik-Khalil, Stephen Taylor, Oliver G. Pybus, Barbro Mäkitalo, Lilian Walther-Jallow, Mats Spångberg, Rigmor Thorstensson, Adnane Achour, Eva Maria Fenyö, Guillaume B. E. Stewart-Jones, Anna-Lena Spetz

**Affiliations:** 1 Department of Laboratory Medicine, Lund University, Lund, Sweden; 2 Human Immunology Unit, Weatherall Institute of Molecular Medicine, John Radcliffe Hospital, Oxford University, Oxford, United Kingdom; 3 Center for Infectious Medicine, Department of Medicine, Karolinska University Hospital Huddinge, Karolinska Institutet, Stockholm, Sweden; 4 Computational Biology Research Group, Weatherall Institute of Molecular Medicine, John Radcliffe Hospital, Oxford University, Oxford, United Kingdom; 5 Department of Zoology, Oxford University, Oxford, United Kingdom; 6 Swedish Institute for Infectious Disease Control, Stockholm, Sweden; Harvard Medical School, United States of America

## Abstract

Neutralizing antibodies (NAb) able to react to heterologous viruses are generated during natural HIV-1 infection in some individuals. Further knowledge is required in order to understand the factors contributing to induction of cross-reactive NAb responses. Here a well-established model of experimental pathogenic infection in cynomolgus macaques, which reproduces long-lasting HIV-1 infection, was used to study the NAb response as well as the viral evolution of the highly neutralization-resistant SIVmac239. Twelve animals were infected intravenously with SIVmac239. Antiretroviral therapy (ART) was initiated ten days post-inoculation and administered daily for four months. Viral load, CD4^+^ T-cell counts, total IgG levels, and breadth as well as strength of NAb in plasma were compared simultaneously over 14 months. In addition, *env*s from plasma samples were sequenced at three time points in all animals in order to assess viral evolution. We report here that seven of the 12 animals controlled viremia to below 10^4^ copies/ml of plasma after discontinuation of ART and that this control was associated with a low level of evolutionary divergence. Macaques that controlled viral load developed broader NAb responses early on. Furthermore, escape mutations, such as V67M and R751G, were identified in virus sequenced from all animals with uncontrolled viremia. Bayesian estimation of ancestral population genetic diversity (PGD) showed an increase in this value in non-controlling or transient-controlling animals during the first 5.5 months of infection, in contrast to virus-controlling animals. Similarly, non- or transient controllers displayed more positively-selected amino-acid substitutions. An early increase in PGD, resulting in the generation of positively-selected amino-acid substitutions, greater divergence and relative high viral load after ART withdrawal, may have contributed to the generation of potent NAb in several animals after SIVmac239 infection. However, early broad NAb responses correlated with relatively preserved CD4^+^ T-cell numbers, low viral load and limited viral divergence.

## Author Summary

In a longitudinal study of clinical and evolutionary responses to transient treatment in 12 experimentally-infected macaques, subjects show clear stratification into two groups based on viral load, immunological response, and evolutionary factors. Subjects that controlled viremia following withdrawal of treatment developed broadly neutralizing antibody responses earlier than subjects with no or transient control of viremia. Moreover, this latter group of macaques with higher viral loads showed greater divergence of SIV sequences, greater numbers of positively-selected amino-acid substitutions and a stronger neutralizing antibody response. The increase in viral genetic diversity started at an early stage of infection. The authors propose that this early phase of evolution is principally responsible for the later failure to control viremia and resulted in the development of potent neutralizing capacity.

## Introduction

The design of an HIV-1 envelope (Env) immunogen capable of inducing broadly reactive neutralizing antibodies (NAb) has so far proven extremely difficult. Although NAb directed against Env can be detected early in infection in a majority of patients, this antibody response is generally not able to neutralize heterologous viruses [1]–[4]. However, some HIV-1 infected patients eventually develop broadly reactive NAb capable of neutralizing several different viral isolates [5]–[9]. Such development of NAb responses has been associated with long-term non-progression [10]–[12], whilst loss of neutralizing activity has been associated with progression of disease [12], [13]. Although the relative contribution of NAb to prevent progression to AIDS is still unclear [6], [7], the induction of broadly NAb directed against HIV-1 through vaccination is considered to represent a milestone for the development of HIV-1 vaccines [14]. This view is supported by proof-of-concept studies that demonstrated protection against simian immunodeficiency virus (SIV) with a human immunodeficiency virus type 1 envelope (SHIV) through the passive administration of antibodies with cross-neutralizing capacity [15]–[23]. Recently, broad and potent (high-titer) NAb were identified in HIV-1 infected individuals and an analytical selection algorithm for characterization of the NAb response was provided [9]. However, further knowledge is still required to understand how the immune system may generate cross-reactive NAb responses against HIV-1. Factors contributing to the elicitation of broadly NAb may include the magnitude and duration of viral replication, the preservation of CD4^+^ T cells, the degree of B-cell depletion, the conformation of Env during primary infection, or the appearance of certain envelope structures during infection [14]. Addressing these factors in patients is difficult as the sequence of the founder virus and the time and dose of infection are usually unknown. In addition, patients can be infected with different strains of viruses [24].

The SIV/SHIV macaque model has been extensively used as a surrogate for HIV-1 infection to study pathogenesis, and test vaccine candidates or novel therapeutics [25]. Infection with a characterized strain of SIV enables studies of disease progression in conjunction with the viral evolution and generation of antibody responses in macaques. Inoculation with SIV or SHIV viruses can result in induction of NAb. However the high degree of viral diversity generated *in vivo* very often leads to antigenic escape variants and a high replication rate in the macaques [26]–[28].

Earlier studies have described the evolution of SIV by the use of comparative techniques; essentially quantifying amino-acid substitutions in small numbers of viruses cloned from different individuals and compared to a consensus sequence [29]. However, it has since become clear from longitudinal studies of within-host HIV-1 [30] and hepatitis C virus [31] evolution that key evolutionary parameters as measured at the within-host level (for instance evolutionary rate) differ from estimates obtained at the host-population level (by sampling different individuals). Thus, better understanding of HIV/SIV evolution strongly highlighted the importance of sampling viral diversity over time as well as in different hosts in order to accurately describe viral sequence evolution.

Furthermore, previous comparative studies of consensus sequences [32] ignored the loss of statistical independence due to shared phylogenetic ancestry [33]. Thus, viral genetic changes observed among closely-related taxa may represent non-beneficial mutations that have yet to be filtered out by selection, rather than key adaptive mutations. However, recently improved phylogenetic methods allow inference of the strength of positive (diversifying) and negative (purifying) selection [34] on a site-wise basis as well as to identify selection pressure variations within genes in several viruses [35].

Here we have used experimental pathogenic infection in cynomolgus macaques, a well-established model for long-lasting HIV-1 infection, in order to study the appearance of NAb as well as to follow the evolution of the viral population. Twelve cynomolgus macaques were infected with SIVmac239 and subjected to early antiretroviral therapy (ART). Early ART has previously been demonstrated to preserve SIV/HIV-specific cellular immune responses, which may be beneficial for long-term control of viremia [36]–[38]. However, less is known about the emergence of NAb responses following early ART. As depletion of CD4^+^ T cells occurs early following infection with SIVmac239 [39], treatment with tenofovir was initiated ten days after viral inoculation. Thereafter ART was provided between 10 days and four months post-inoculation. We monitored plasma viremia, CD4^+^ T-cell counts and NAb titers throughout the 14 month study period. In addition, we studied the viral evolution using a total of 281 full-length *env* sequences obtained over the course of the study from plasma samples and viral re-isolates as well as the inoculate virus.

We demonstrate that early single drug treatment effectively controlled viremia in nearly all animals (11 out of 12). In addition, a majority of animals (seven out of 12) maintained good control of viremia even after therapy withdrawal (defined as below 10^4^ viral copies post-ART throughout the study). Interestingly, the five macaques that failed to control viremia following ART withdrawal acquired the V67M and R751G mutations previously reported to occur in viral escape variants in a rhesus macaque that developed unusually high titers of NAb against SIVmac239 [40]. We also report the induction of high NAb titers in all 12 cynomolgus macaques following infection with SIVmac239 and early treatment with ART. The strength of the NAb response was greater in the macaques with poor control of viral load, greater divergence in *env* and higher numbers of positively-selected sites early in infection. We therefore conclude that the increase in viral population genetic diversity, which occurred prior to the increase in viral load after ART withdrawal, contributed to the overall strength of the NAb response.

## Results

### A majority of SIVmac239 infected cynomolgus macaques subjected to early short-term ART efficiently control viremia

**Figure 1:**
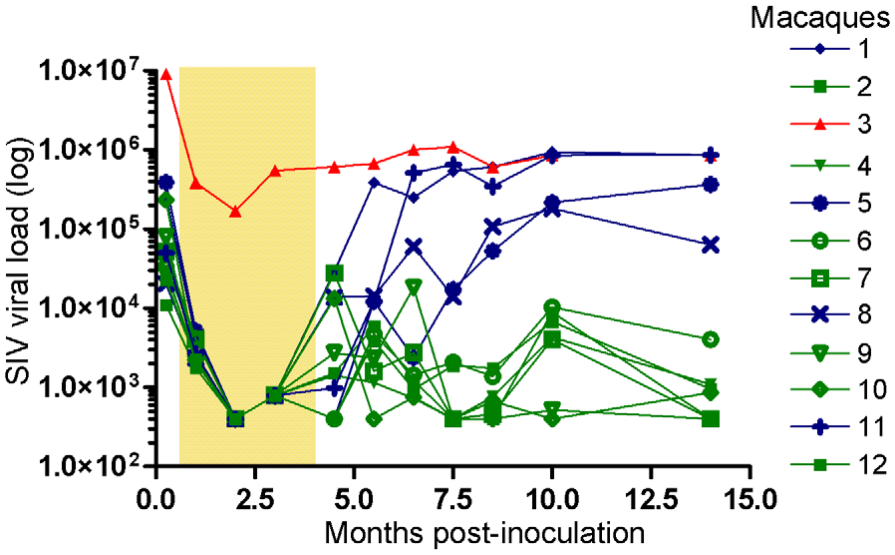
Viral loads in macaques after SIVmac239 infection. Twelve 4-year-old male Cynomolgus macaques (*Macaca fascicularis*) were infected intravenously with pathogenic cell-free SIVmac239. Treatment with tenofovir was started ten days after inoculation and provided by subcutaneous injections for 16 weeks, as indicated by the shaded area. Viral loads are depicted longitudinally for each animal. Animals 2, 4, 6, 7, 9, 10 and 12 were designated longterm controllers (LC, green), as defined by maintained viral load below 10,000 copies/ml after treatment interruption. Animals 1, 5, 8 and 11 were designated transient controllers (TC, blue) due to progressive increase of viral load following treatment interruption and animal 3 non-controller (NC, red) due to persistent high (over 100,000 copies/ml) viral replication throughout the study. Statistically significant differences were observed between the LC and NC/TC groups with regards to viral load starting from 4.5 months after inoculation (*p* = 0.002, Mann-Whitney non-parametric test and *p*<0.0001, two-way repeated measures analysis of variance model).

Twelve animals were inoculated intravenously with SIVmac239. Following confirmation of infection, all animals received daily subcutaneous injections of tenofovir, starting on day 10 for a period of four months (Figure 1). Plasma viral load, CD4^+^ T-cell counts and plasma samples for measuring NAb were collected on 11 occasions during the 14 month study period (Figure 1, Table 1 and Figure S1). One animal (number 3), which was the smallest in the group, displayed a high viral load of 9 million copies/ml seven days after inoculation. Treatment with tenofovir resulted in a 50-fold reduction in viral load, which was maintained at around 400,000 copies/ml of plasma throughout the study period. This animal was therefore referred to as non-controller (NC 3). In all other macaques, viral load fell below the limit of detection of the viral load assay during tenofovir treatment (Figure 1). Seven macaques (numbers 2, 4, 6, 7, 9, 10 and 12) were designated long-term controllers (LC) as they maintained a plasma viral load of below 10,000 copies/ml following discontinuation of treatment. The remaining four macaques (numbers 1, 5, 8 and 11) displayed a progressive increase in viral load post-treatment, with levels ranging from 10,000 to 1 million copies/ml of plasma and were thus designated as transient controllers (TC). Statistically significant differences in viral load were observed between the LC and TC groups starting at 5.5 months after inoculation (*p* = 0.008, Mann-Whitney non-parametric test and *p*<0.0001, two-way repeated measures analysis of variance model).

**Table 1:** CD4^+^ T-cell changes and IgG content in plasma over time.

Macaque	Group*	CD4^+^ T-cell change (%)†			Plasma IgG change (%)‡			
		7 days p.i.	During therapy	After therapy	During therapy	5.5–6.5 months p.i.	7.5–8.5 months p.i	10–14 months p.i.
**3**	**NC**	−26.2	−11.6	−51.5	24.8	80.2	44.7	7.90
**1**	**TC**	−33.1	−9.30	−53.0	−19.0	9.40	−6.20	−25.6
**5**	**TC**	−34.5	−37.1	−52.2	14.7	26.4	29.3	37.4
**8**	**TC**	−20.9	10.7	−15.0	10.7	50.6	10.4	9.0
**11**	**TC**	−50.5	2.10	−24.9	11.8	14.2	38.0	27.6
**2**	**LC**	20.0	−7.40	−39.2	5.20	−4.30	1.80	−14.3
**4**	**LC**	−33.0	3.20	−30.2	7.70	−25.3	−19.2	−8.10
**6**	**LC**	−46.9	−28.6	−39.7	3.0	13.4	−17.7	−31.0
**7**	**LC**	−8.10	12.4	−9.90	−6.40	−6.3	−13.5	−19.0
**9**	**LC**	−37.2	−19.1	−38.8	−4.10	−1.4	−8.1	−26.3
**10**	**LC**	−41.2	−21.8	−5.0	21.3	14.2	−0.3	1.80
**12**	**LC**	17.9	23.1	−18.2	19.8	18.2	12.4	−7.80

*NC: non-controller, TC: transient controller, and LC: long-term controller.

**†:** CD4^+^ T-cell change: was calculated by using the mean value of CD4^+^ T-cell counts in a defined period and is expressed as a percentage of CD4^+^ T-cell numbers before inoculation.

Mean of three samples were used during the therapy phase and seven samples after therapy.

**‡:** Plasma IgG change: was calculated by using the mean value of IgG in a defined period and is expressed as a percentage of IgG before inoculation.

Mean of three samples were used during the therapy phase and thereafter two samples for each time period.

A marked decline in CD4^+^ T-cell counts (more than 20%) was detected in the peripheral blood seven days after inoculation in all animals except for macaques 2, 7 and 12, later classified as LC (Table 1). The decline of CD4^+^ T cells generally slowed following initiation of tenofovir therapy; CD4^+^ T-cell counts reached pre-infection levels in some macaques (Figure S1). Nevertheless, upon therapy interruption, gradual CD4^+^ T-cell decline occurred over time and was most pronounced in the NC/TC macaques. A significant difference (*p*<0.0001) in CD4^+^ T-cell counts was found between the LC and NC/TC groups starting at week 22, which corresponds to four weeks without tenofovir (two-way repeated measures analysis of variance model).

Hypergammaglobulinemia is a known hallmark of HIV-1 infection and therefore plasma IgG levels were measured relative to baseline IgG (Table 1). IgG levels peaked with an 80% increase at 5.5–6.5 months after inoculation in macaque NC3. Three of the TC macaques (5, 8, 11) also displayed an increase in IgG levels with peaks of 37–50% above baseline at 5.5–8.5 months post-inoculation. Neither the LC macaques nor the macaque TC1 displayed markedly elevated IgG levels (>30%) throughout the study period. After treatment interruption, a significantly greater increase in plasma IgG level was detected in NC/TC macaques compared with LC macaques (*p* = 0.018, Mann-Whitney non-parametric test).

### The magnitude of sequence divergence correlates to viral control

**Figure 2:**
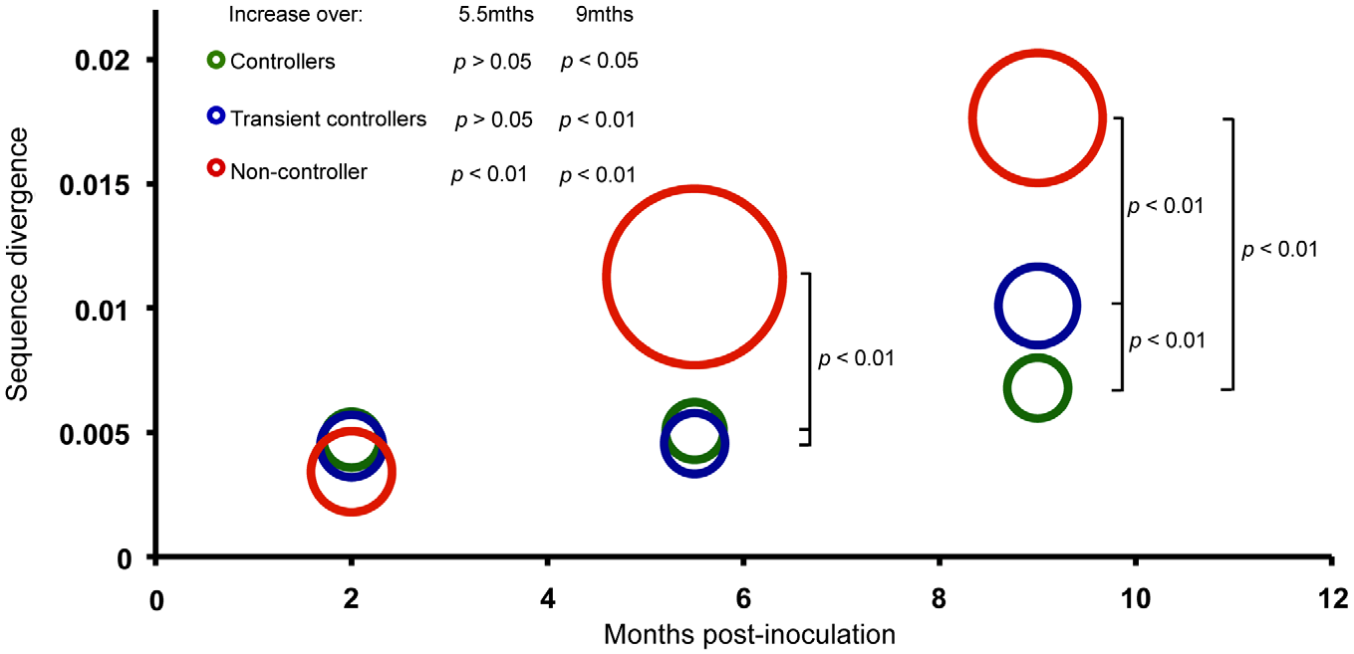
Sequence divergence from SIVmac239 in controller, transient-controller and non-controller macaques. Full-length *env*s were isolated and sequenced from 12 macaques at 2, 5.5 and 9 months p.i. with SIVmac239. The level of sequence divergence from the inoculate strain was estimated from the phylogeny under the CTR+γ+I model. For each group of macaques (LC, TC and NC), the mean of divergence (position of circle centre on y axis) and the mean of diversity (radius) is represented here. Mean divergence increased significantly over time in all macaque groups (5.5 months: LC *p*>0.05, TC *p*>0.05, NC *p*<0.01; 9 months: LC *p*<0.05, TC *p*<0.01, NC *p*<0.01) as determined by two-sample T-tests (with Welch approximations for degrees of freedom). Mean divergence differed significantly between groups of macaques at 5.5 months (LC vs NC: *p*<0.01; TC vs NC: *p*<0.01) and 9 months (All groups: *p*<0.01) p.i. Sequences from macaque NC3 were significantly more diverse than those of LC and TC macaques at the 5.5 (*p*<0.05) and 9 (*p*<0.01) month time points.

To study SIV evolution, a total of 281 full-length *env* sequences were obtained, with a mean of seven sequences per plasma time point (2, 5.5 and 9 months p.i.; range 2–10) (Table S1) and eight sequences from the inoculate virus. The level of sequence divergence between the inoculate strain SIVmac239 and each analysed sequence was estimated from the phylogeny (units of expected substitutions per site). Divergence, therefore, is a measure of how different a given analyzed sequence is from the parental strain. Mean site-wise Shannon entropy was used to measure sequence diversity for each macaque at the three time-points. Macaques were grouped according to their viral control (NC, TC, LC), and the mean divergence (position of circle on y-axis) and diversity (size of circles) calculated (Figure 2). The mean divergence increased significantly over time in the NC macaque (*p*<0.01 at both 5.5 and 9 months) while the mean divergence in TC and LC macaques was significantly greater at 9 months (*p*<0.01 and *p*<0.05, respectively). Significant differences were also observed between the animal groups at both the 5.5 and 9 month time points (*p*<0.01), with macaque NC3 displaying the greatest divergence when compared to the two other groups. Additionally, mean divergence of TC macaques from SIVmac239 was greater than LC macaques at nine months (*p*<0.01). Sequences from macaque NC3 were significantly more diverse than those of LC and TC macaques at the 5.5 and 9 month time points (*p*<0.05 and *p*<0.01 respectively).

### High magnitude neutralizing antibody responses detected in macaques with high viremia

**Figure 3:**
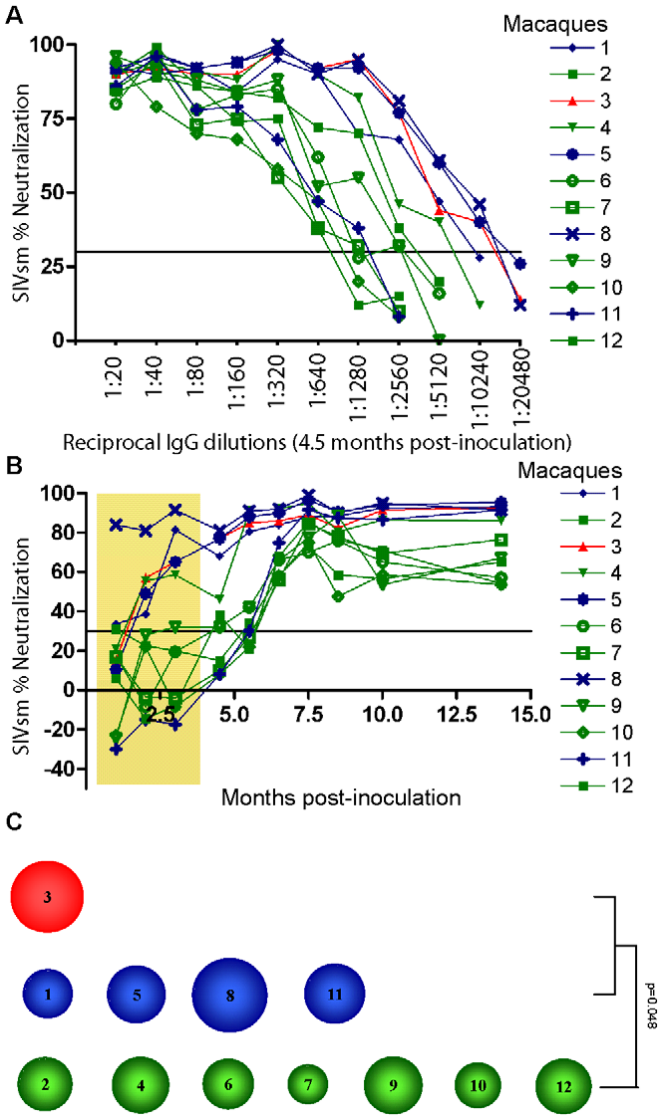
Heterologous neutralization of SIVsm. (A) Purified IgG obtained from plasma samples 4.5 months post-inoculation were titrated and analyzed for neutralization of SIVsm (SMM-3). Neutralization profiles of NC (animal 3, red) and TC (animals 1, 5, 8, 11, blue) as well as LC (animals 2, 4, 6, 7, 9, 10, 12, green) are shown. Values are means of two independent assays. Assay cut-off was 30% as indicated by line. The NC/TC macaques displayed a significantly greater magnitude of neutralization against SIVsm compared with the LC group (*p* = 0.043, Mann-Whitney test). (B) Purified IgG corresponding to 1∶2560 dilution were analyzed for neutralization of SIVsm in samples obtained at ten different time points. Tenofovir treatment period is indicated by shaded area. (C) The potency score for each macaque was calculated by summarizing the magnitude (neutralization score) against each virus and then dividing by total number of viruses tested (n = 8) as shown in Table 2. The magnitude of an individual IgG sample was determined by its neutralization score defined as log-transformed titers. Log-transformed titers were calculated by dividing the highest neutralizing titer values by 100 before applying a log-base 3 transformation and then adding 1 [Y = log3 (dilution/100)+1]. The size of circle corresponds to the potency score. The NC/TC macaques displayed a significantly greater potency score compared with the LC group (*p* = 0.048, Mann-Whitney test).

SIVmac239 is considered to be neutralization-resistant. Therefore purified IgGs from all 12 macaques were first tested for neutralization of a SIVsm (SMM-3) strain originally isolated from a naturally infected sooty mangabey monkey, reported as neutralization-sensitive [41]–[43]. The magnitude of responses was tested by titrating IgG obtained from the 4.5 month post-inoculation (p.i.) plasma samples. This study revealed high-titer neutralization against SIVsm (up to 1∶10240) (Figure 3A). The highest titers against SIVsm were detected in macaques NC3 and TC1, 5, 8 (≥1∶5120) while the LC group displayed lower titers (ranging from 1∶640 to 1∶5120). High–titer neutralization against the SIVsm-related HIV-2 was also detected (Figure S2A and S2B).

A plasma dilution of 1∶2560 was chosen to evaluate the kinetics of emergence of NAb against SIVsm. This study revealed an early response (2 months p.i.) already detectable during the treatment phase in NC/TC macaques 3, 1, 5, 8 and in LC number 4 (Figure 3B). The remaining animals neutralized SIVsm at a titer of 1∶2560 starting from 5.5 months after inoculation. The NC/TC macaques displayed a significantly greater magnitude of neutralization against SIVsm compared with the LC group (*p* = 0.043, Mann-Whitney test). IgG from the 4.5 month plasma samples was also titrated against two additional SIVsm isolates, one known to be highly neutralization sensitive (SIVsm:C39^sens^) and the other resistant to neutralization (SIVsm:C39^res^) [43]. As expected, a majority of macaques had higher titers against SIVsm: C39sens than SIVsm: C39res (Table 2). The two highest titers against the neutralization resistant virus were obtained with IgG (from NC3 and TC8) that strongly neutralized the SIVsm stock as well. Plasma IgG from all 12 macaques was also titrated against SIVmac239 and SIVmac251 as well as two HIV-2 isolates and 5 HIV-1 isolates. These studies showed that NAb against both HIV-2 isolates were present at high titers, whilst the capacity to neutralize HIV-1 CC30 was achieved with 1∶20–80 dilutions, similar to the titers against SIVmac239 inoculate virus and SIVmac251 (Table 2). Neutralization against the other HIV-1 isolates SF162, 92UG024, 92Br025 and CC48 were only achieved with 1∶20 dilutions in certain animals (data not shown). The mean of magnitudes of neutralization was calculated by log transforming the titers according to the formula described in [9] and applied for the panel of 8 viruses in Table 2. The NC/TC macaques with high level of viremia displayed a more potent NAb response compared with the LC macaques (*p* = 0.048, Mann-Whitney test) (Table 2 and Figure 3C).

**Table 2:** Neutralization profiles.

			SIV					HIV-2		HIV-1
Macaque	Group*	Potency†	SIVsm: SMM-3	SIVsm:C39sens	SIVsm:C39res	SIVmac239	SIVmac251	1682	1812	CC30
**3**	**NC**	**4.17**	10240	163840	327680	80	80	10240	10240	20
**1**	**TC**	**2.79**	5120	1280	1280	<20	<20	10240	20480	<20
**5**	**TC**	**3.28**	10240	10240	1280	40	20	40960	20480	20
**8**	**TC**	**4.25**	10240	20480	327680	40	<20	163840	20480	80
**11**	**TC**	**3.38**	1280	81920	640	40	<20	81920	20480	80
**2**	**LC**	**3.02**	640	40960	1280	20	<20	40960	10240	20
**4**	**LC**	**3.20**	5120	10240	1280	40	20	81920	10240	20
**6**	**LC**	**2.86**	2560	40960	1280	20	20	5120	5120	20
**7**	**LC**	**2.23**	1280	1280	320	20	20	2560	10240	20
**9**	**LC**	**3.26**	2560	81920	640	20	20	81920	10240	20
**10**	**LC**	**2.57**	640	1280	1280	<20	<20	5120	20480	80
**12**	**LC**	**3.14**	2560	40960	640	40	20	81920	2560	80

*NC: non-controller, TC: transient controller, and LC: long-term controller.

The titers correspond to 30% neutralization. The magnitude of response for each macaque IgG is calculated by; log 3(titer/100)+1.

**†:** The potency is mean of the magnitudes for each macaque against the given panel of SIV, HIV-2 and HIV-1 viruses.

The NC/TC macaques with high level of viremia displayed a more potent NAb response compared with the LC macaques (p = 0.048, Mann-Whitney test).

### Broad early neutralizing antibody responses in macaques with controlled viremia

**Figure 4:**
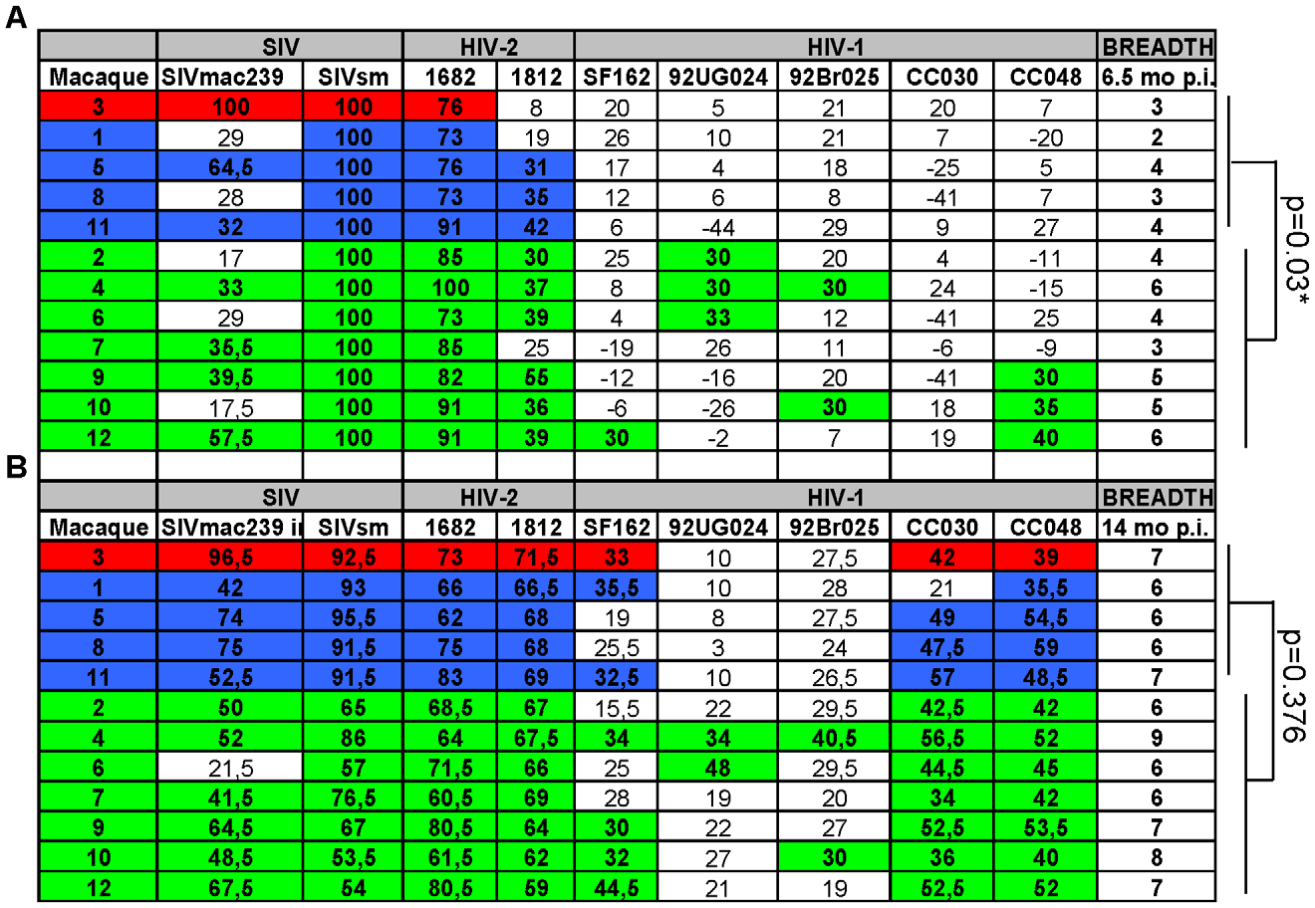
Evolution of neutralization breadth over time. Comparison of neutralization breadth at 6.5 (A) and 14 (B) months post-inoculation. Color code: NC, red, TC, blue, LC, green Figure denotes % neutralization at 1∶20 dilution. SIVsm used was SMM-3. The LC macaques displayed a significantly greater breadth at 6.5 months compared with the NC/TC group (*p* = 0.03, Mann-Whitney test).

Breadth of neutralization was defined as the number of viruses neutralized at a titer of 20 in purified IgG samples using the cut-off ≥3SD in the standardized neutralization assay. Nine viruses were included in the panel and IgG from all 12 animals obtained at 6.5 and 14 months p.i. were assessed (Figures 4A and 4B, respectively). LC macaques displayed an early broad neutralization response that was significantly higher compared with the NC/TC group at 6.5 months after inoculation (Figure 4A, *p* = 0.03 Mann- Whitney test). The number of virus isolates neutralized ranged from 2–6 at this time point. However, the breadth of responses increased over time in all animals and a significant difference was no longer detectable at 14 months p.i. (Figure 4B). The broadest NAb response was detected in macaque LC4 which neutralized all nine viruses tested. The results indicate an association between control of viremia and development of a broad NAb response early in infection.

### Emergence of neutralization resistant viruses and potent antibody maturation in macaques NC3 and TC1

**Figure 5:**
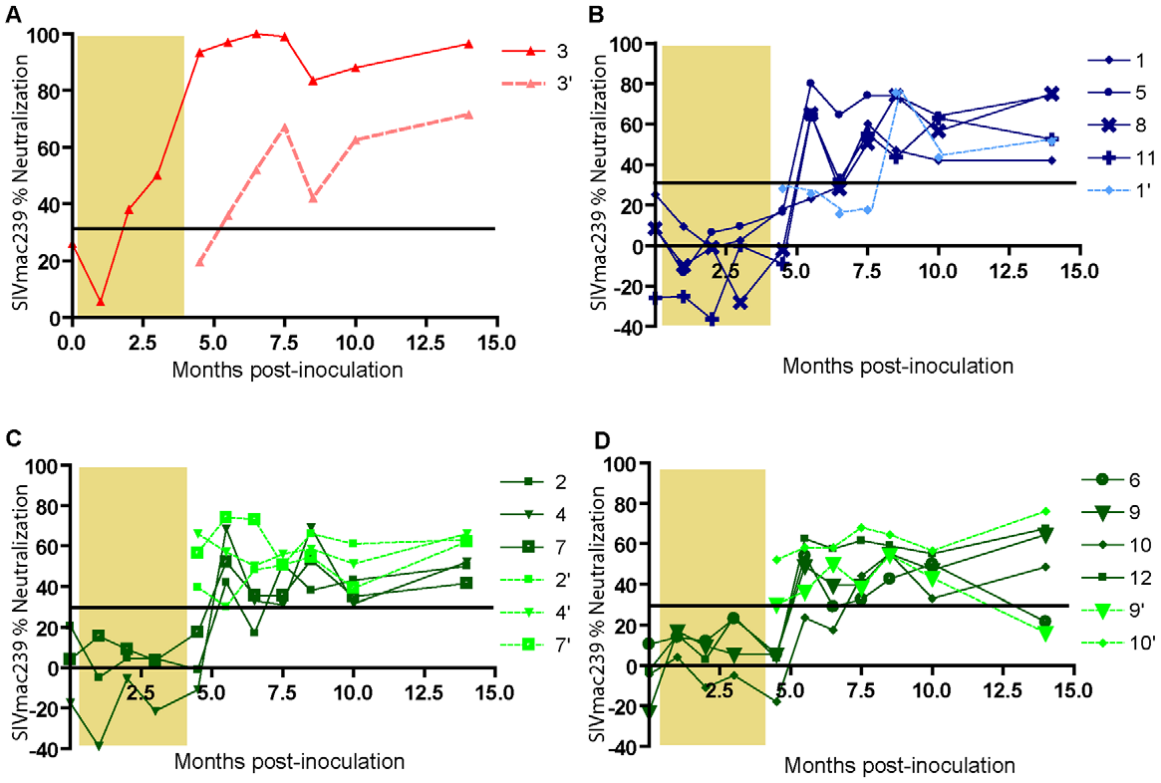
Autologous neutralization of SIVmac239. Purified IgG corresponding to 1∶20 dilutions were analyzed for neutralization of SIVmac239 in samples obtained at eleven different time points. Tenofovir treatment period is indicated by shaded area. (A) Development of neutralizing plasma titers against SIVmac239 inoculate in NC3 (3; red) and the 1^st^ re-isolate (3′; pink), (B) in TC macaques against inoculate (dark blue) and reisolates (′; light blue). (C) and (D) Neutralization against inoculate in LC animals (dark green) and reisolates (′; light green). Values are means of two independent assays. Assay cut-off was 30% as indicated by line.

Re-isolation of virus from all animals was attempted at 4.5 months p.i. (two weeks after ART withdrawal) as well as at 9 months p.i. and the NAb response of the purified IgG was measured against the autologous and inoculate virus in order to follow the evolution of viral neutralization resistances and neutralization capacity (Figure 5).

The 4.5 month re-isolates from macaques NC3 and TC1 were resistant to autologous neutralization (at 5.5 months) although their IgG effectively neutralized the parental virus SIVmac239 (Figure 5A and 5B). This suggests the emergence of neutralization resistant variants in these animals. Sequences from re-isolated viruses (4.5 months p.i.) derived NC3 were therefore compared to the SIVmac239 inoculate sequence in order to identify potential amino acid substitutions. In all four clones derived from NC3, a variety of mutations occurred, including V67M, A417T and R751G (Figure S3). In contrast, all five re-isolates obtained at 4.5 months p.i. from the LC macaques were neutralized by the corresponding autologous IgG, even though no neutralizing activity against the inoculate SIVmac239 could be detected before 5.5 months in these macaques (Figure 5C and 5D). This could be explained by the early development of broad NAb responses in LC macaques as compared to the NC/TC group.

IgG from 8.5–10 months p.i. samples from macaque NC3, neutralized both the 4.5-months and 9-months re-isolates. Similarly, the 4.5-months re-isolate from macaque TC1 was neutralized by IgG obtained from later samples. A likely explanation for this is affinity maturation of NAb that takes place over time. It is also possible that the increase in breadth of NAb response over time has contributed to neutralization of these re-isolates. It should be noted that this increase of neutralization capacity did not result in a significant reduction in viral load in either of the macaques (Figure 1), suggesting that selection of neutralization resistant variant viruses is a continuous process [1].

### Site wise selection analyses show large numbers of positively-selected mutations in NC and TC animals that occur early in infection

To differentiate between amino-acid substitutions arising from neutral evolution and those arising that confer a selective advantage, we estimated the ratio of non-synonymous to synonymous nucleotide changes (dN ∶ dS) at individual codons. The site-wise (dN/dS) analyses were carried out as detailed in the Methods and corrected for multiple testing using the Benjamini & Hochberg [44] false discovery method with a critical value of 0.05. Following correction, 39 sites were found to be under significant positive (diversifying) selection, where there is an excess of non-synonymous nucleotide changes. A paucity of nucleotide changes resulting in codon substitution where synonymous changes were plentiful suggested a constrained (negatively-selected) site; 49 codons were found to be under significant negative (purifying) selection at alpha = 0.05 (Table S2).

**Figure 6:**
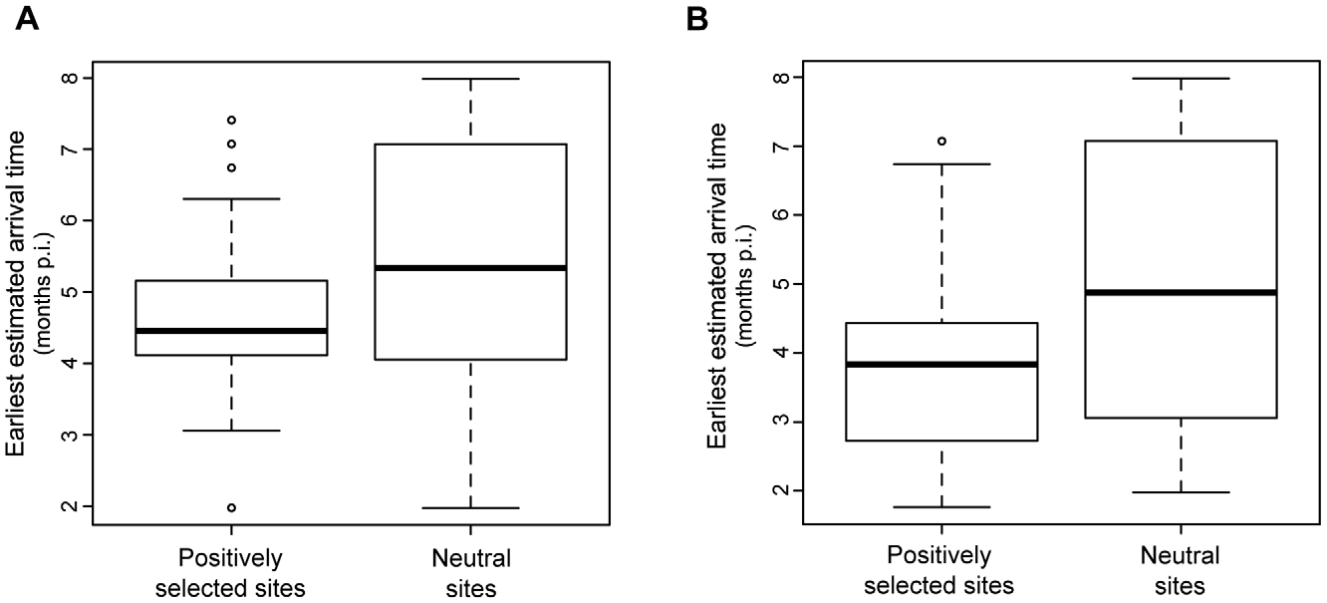
Estimated arrival times of amino-acid substitutions. Amino-acid substitutions were tracked through the whole phylogeny by estimating ancestral sequences at every internal node. The elapsed time since infection and genotypes of these ancestral sequences were then compared to estimate the set of first occurrences in any given lineage (‘arrival times’) for each substitution. (A) Box plot of estimated earliest (minimum) arrival times; (B) Box plot of estimated latest (maximum) arrival times. Sites with *p* (dN/dS>1)≤0.05 (see Methods) were categorised as ‘positively-selected.’ Substitutions at other sites were therefore assumed to have occurred neutrally.

The existence of chains of mutations whose sequential arrival is replicated in separate individuals is predicted to be a consequence of evolutionary responses by the virus to selection pressure from lymphocyte activity (‘escape’ mutations). In order to gain insights into these sequential amino-acid substitutions, particularly escape mutations, we took advantage of temporal information in the phylogeny to reconstruct the earliest time at which each amino-acid substitution occurred, defined here as the ‘arrival time’ of a substitution. We therefore compared the estimated arrival time for all sites predicted to have significantly positively-selected amino-acid substitutions with the arrival time of appearance for amino-acid replacements that were not significantly likely to represent positively-selected sites. A one-way ANOVA determined that the mean time of first appearance was earlier for positively-selected substitutions compared to neutrally-selected substitutions. Furthermore, we observed that positively-selected substitutions tended to occur during or very shortly after the treatment period (Figure 6). When the earliest estimated arrival of positively-selected sites are mapped to the phylogeny (Figure S4), it can clearly be observed that LC macaque sequences exhibit far fewer positively-selected substitutions than those of NC/TC macaques.

### Early increase of viral population genetic diversity detected in non-controller macaques

In the presence of selection, there is a non-linear relationship between the absolute size of the viral population (as measured by viral load) and the population's genotypic diversity. For example, a large population may be less diverse than a smaller one, potentially exposing the host immune system to a smaller variety of antigens [45]. We therefore estimated ancestral population genetic diversity (PGD) through time. These PGD plots, or ‘skyline’, plots provide a better indication of the variety of antigens likely to have challenged the host immune system than diversity or absolute population size alone. As described in the Methods, substitution model and mean evolutionary rate parameters were jointly estimated from the combined data set, since some individual animals' data sets did not contain sufficient sampling intensity to allow these parameters to be separately estimated. Post-hoc rate correction to allow for heterogeneous mean substitution rates among animals was achieved by scaling the estimated ancestral PGD plots such that individual animals' tree mean posterior root heights were equal.

**Figure 7:**
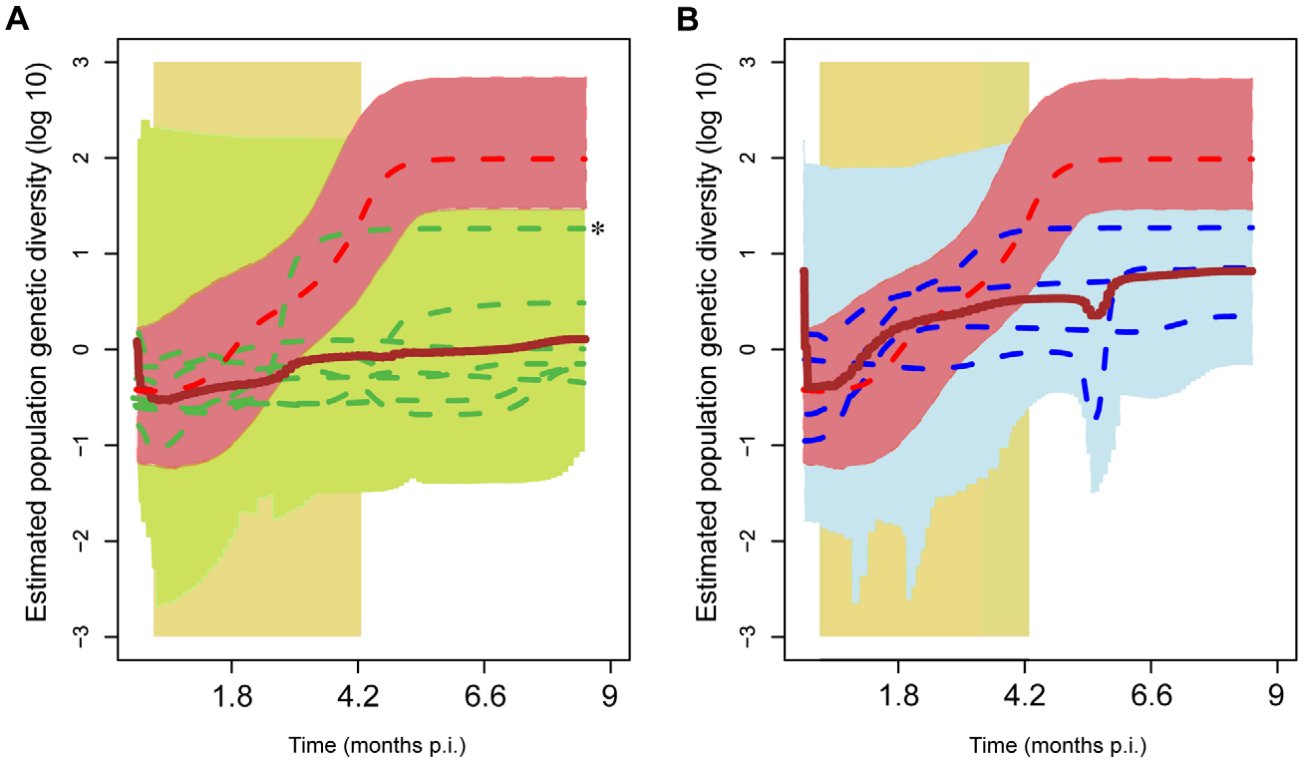
Viral population genetic diversity through time (‘skyline plots’) of individual infections. Skyline plots were obtained through BEAST and show the estimated viral population genetic diversity (PGD) through time. (A) Median posterior PGD of LC animals (2, 4, 6, 7, 9, 10 & 12; dashed green lines); mean median PGD of the set of all LC animals (brown line) and maxima and minima of upper and lower 95% confidence intervals for the set (shaded green area). A much larger median posterior PGD was estimated for animal 10 than for the other LC animals; this is marked with an asterisk (*). However the 95% confidence intervals for this animal were also largest in the LC group. (B) Median posterior PGD of TC animals (1, 5, 8 & 11; dashed blue lines); mean median PGD of the set of all TC animals (brown line) and maxima and minima of upper and lower 95% confidence intervals for the set (shaded blue area). Both plots: NC (animal 3) median PGD (thick red line) and upper and lower 95% confidence intervals (shaded red area). The shaded yellow area covers the treatment period. In all plots post-hoc rate correction was employed to scale the PGD on the time axis (see Methods).

The individual traces for each animal can be seen in Figures 7A and 7B, which show the PGD trace for the NC3 macaque superimposed with the mean posterior PGD for TC and LC groups respectively. The 95% confidence intervals on population size are shown by the upper and lower bounds on the PGD traces, while the lower 95% confidence interval on root height (time of most recent common ancestor) is shown by vertical dashed lines. It can clearly be seen that LC macaques (2, 4, 6, 7, 9, 10, 12) experience persistently lower and more slowly increasing population diversity than both TC macaques (1, 5, 8 & 11) and the NC (macaque 3). Furthermore, while PGD levels were held low during the treatment period for LC macaques (and viral *loads* decreased during treatment for *all* animals, except NC3), the data suggest that PGD for TC macaques began to increase before the end of treatment (however we cannot unambiguously resolve the timing of the PGD increase due to the large confidence intervals on these estimates.) The NC3 experienced persistently high and increasing population genetic diversity throughout the study.

### The positively-selected mutations are localized in different regions of gp120 in NC, TC and LC macaques

Positively-selected (Table S2) mutations were analyzed for their appearance in the three different groups of macaques. Substitutions V67M, A417T, R751G and L802F appeared in macaque NC3 and in several TC macaques. In contrast none of the LC-derived viruses carried any of these substitutions. Mutation A417T results in an additional glycosylation site in V4 domain, previously found to result in a dramatic increase in neutralization resistance [40]. Interestingly the L802F, R751G, and V67M substitutions are associated with increased replicative capacity and are also present in viruses adapted to macrophages or isolated from the CNS of infected macaques [46], [47].

**Figure 8:**
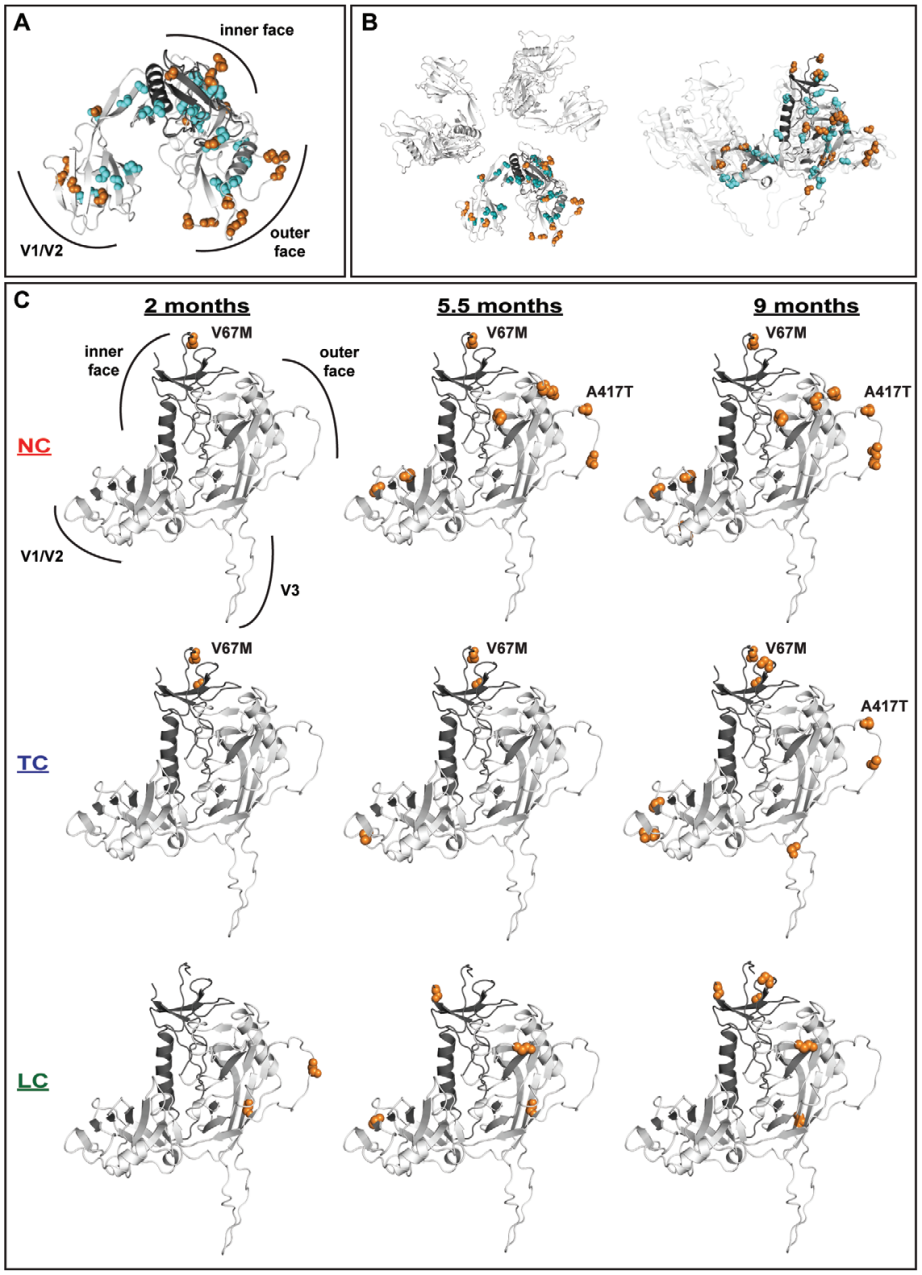
Distribution of significantly selected mutations on gp120. Mutated sites were mapped on a molecular model of SIV gp120. The inner domain of the monomer is displayed in dark grey. Positively- and negatively-selected mutations are highlighted as orange and cyan spheres, respectively. (A) Combined analysis of the localization of the positively- and negatively-selected mutations found at 2, 5.5 and 9 months. The gp120 monomer is oriented with the V3 domain pointing away from the viewer. In contrast to the conserved sites that are mostly localized within the core of the monomer, the positively-selected mutations are predominantly exposed on the surface of gp120. (B) The selected mutations are highlighted in a molecular model of the gp120 trimer viewed with the same orientation as in 8A (left) or ‘from the side’ following a 90° rotation (right). (C) Progressive and clustered appearance of the positively-selected mutations analyzed individually for each of the three different monkey groups LC, TC and NC at 2, 5.5 and 9 months. The inner and outer as well as the V1/V2 and V3 domains are indicated. The orientation of the monomer was slightly tilted with respect to (B) and the occurrences and localizations of the V67M and A417T mutations are highlighted.

Statistical support for positive selection was compared between the entire gene and each hypervariable (V) region in gp120. Strongest support for evolutionary pressure was found in V1, V2 and V5, with significantly higher eCDF values at *p*<0.001 and *p*<0.05 for V2 (Figure S5). Evolutionary linkage (epistasis) of positively-selected sites was investigated using a Bayesian graphical model (Table S3). Analysis of structural proximity in monomeric and trimeric molecular models of SIV gp120 revealed that the identified epistatic interactions were not co-localized (data not shown). Mapping of the selected sites on a molecular model of the SIVgp120 revealed that positively-selected substitutions tended to occur on solvent-exposed regions, while most of the putative trimerisation interface was unaffected (Figures 8A and 8B). Conversely, the conserved sites including several glycosylation sites, cysteines, and other structurally important residues such as the co-receptor binding site were confined to the core of the monomer.

Substitutions, which we have observed to occur early on and in greater numbers in NC/TC macaques, differ in the location and timing of their appearance on gp120 (Figure 8C). A majority of positively-selected mutations had already appeared by 5.5 months in macaque NC3 (Figure 8C). Most notably for the NC/TC macaques many of the positively-selected sites were clustered on the V1/V2, V4 and V5 loops on the outer face of gp120 known to be important in antibody escape. V67M appeared in a solvent-exposed region of gp120 potentially interacting with gp41. Two additional positively-selected sites (K254 and V260) also appeared in proximity to V67M in both TC and LC macaques. The observed differences in positions of positively-selected mutations further indicate differences in gp120 evolution between the three groups of macaques. In particular, the appearance of V67M and A417T, present in NC/TC macaques, may play an important role in the observed viral load and/or NAb responses.

### Multivariate correlation between evolutionary and clinical factors

In order to determine whether any correlation existed between the sequence evolution and clinical measurements, a multivariate rank sum analysis was performed which compared all variables with each other and identified correlations in the data set. Due to lack of normality in some of the data set we opted to perform a non-parametric analysis of ranked data. The results of the neutralization assays were combined to give ranks for NAb potency, NAb breadth at 6.5 months, and NAb breadth at 14 months. Additional measures included: neutralizing titer against SIVsm, viral load at 6.5 months, and decline of CD4^+^ T cells by 6.5 months as well as CD4^+^ T-cell counts at 6.5 months. Key evolutionary parameters analysed were mean sampled diversity per animal, divergence from the inoculate strain as estimated by maximum likelihood reconstruction, the number of positively-selected amino-acid substitutions observed at *p*<0.05, and the integral of mean estimated PGD over the treatment period as well as the total study period. The correlation table is shown in (Table 3) with positive correlations (following correction for false discovery rate at alpha = 0.05) marked in bold.

**Table 3:** Multivariate correlation between evolutionary and clinical factors.

	Viral Load Mid^a^	Breadth Mid^a^	Breadth Late^b^	Potency^c^	Divergence^d^	Diversity^e^	CD4 Decline^f^	CD4 Count^g^	No. pos sites^h^	Neut SIVsm:SMM-3^i^	PGD Treatment^j^	PGD All^k^
**Viral Load Mid**		0.992	0.287	0.060	0.347	0.764	0.867	0.996	0.010*	0.104	0.662	0.347
**Breadth Mid**			0.426	0.658	0.605	0.183	0.151	0.036*	0.976	0.819	0.486	0.251
**Breadth Late**				0.819	0.819	0.336	0.120	0.353	0.562	0.749	0.059	0.022*
**Potency**					0.033*	0.338	0.727	0.928	0.015*	0.019*	0.610	0.399
**Divergence**						0.066	0.679	0.895	0.116	0.031*	0.364	0.509
**Diversity**							0.778	0.792	0.426	0.621	0.208	0.472
**CD4 Decline**								0.017*	0.914	0.935	0.123	0.463
**CD4 Count**									0.979	0.889	0.222	0.771
**No. pos. sites**										0.012*	0.662	0.523
**Neut SIVsm:SMM-3**											0.685	0.220
**PGD Treatment**												0.338
**PGD All**												

Macaques 1–12 were assigned ranks based on clinical and evolutionary parameters. Corrected *p* values are given where *p*<0.05 indicates statistical significance*.

a Mid = 6.5 months p.i.

b Late = 14 months p.i.

c The potency is the mean of the magnitudes for each macaque against SIV, HIV-2 and HIV-1 shown in Table 2.

d Mean sequence divergence from SIVmac239 over all time points, estimated under CTR+γ+I.

e Mean diversity as determined by mean site-wise Shannon entropy in each subject sequence alignment over all time points.

f Decline in CD4^+^ T-cell numbers from start of study to 6.5 months p.i.

g Total CD4^+^ T-cell count at 6.5 months.

h Total number of positively-selected sites in each subject's sequences.

i NAb SIVsm:SMM-3 titer 4.5 months p.i.

j Integral of PGD during treatment period.

k Integral of PGD over duration of study.

Both sequence divergence and the number of positively-selected mutations correlated with the potency of the NAb response (*p* = 0.033 and *p* = 0.015, respectively). Additionally, a high viral load correlated with a greater number of positively-selected mutations (*p* = 0.010), as previously observed when comparing LC and NC/TC macaques. The NAb SIVsm titer correlated with both sequence divergence (*p* = 0.031) and the number of positively-selected mutations (*p* = 0.012) as well as the overall potency (*p* = 0.019). In terms of the breadth of NAb response, CD4^+^ T-cell count at 6.5 months correlated with the NAb breadth at that time (*p* = 0.036), whilst breadth at 14 months correlated with the PGD over duration of entire study period (*p* = 0.022). The integral of mean PGD during the treatment period was close to being significantly associated with late breadth responses (*p* = 0.059). It therefore appears that LC macaques, with higher CD4^+^ T-cell count, have a broader NAb response at the intermediate time point of 6.5 months but by 14 months the NC/TC macaques, with early high PGD, show greater NAb breadth. Furthermore, the increased sequence divergence observed in the NC/TC macaques correlate with a more potent NAb response than that observed in the LC macaques.

## Discussion

This study represents the most comprehensive longitudinal analyses to date on the appearance of broad and potent NAb responses in the context of viral evolution in 12 experimentally-infected macaques subjected to transient ART. Twelve cynomolgus macaques were inoculated with pathogenic SIVmac239. In order to preserve CD4^+^ T-cell counts, tenofovir was administered daily between 10 days and four months post-inoculation. Eleven out of twelve animals controlled viral loads during treatment. However, on cessation of therapy the transient controller (TC n = 4) group immediately experienced increased viral loads. Not unexpectedly the decline in CD4^+^ T-cell count was tightly correlated to the plasma viral load and was therefore slower in long-term controller (LC n = 7) macaques when compared to NC/TC animals.

A striking feature of our results is a discrepancy between viral population genetic diversity and viral loads over time, in the TC and LC groups. In both TC and LC animals, viral loads were controlled during treatment; only after treatment did their viral loads diverge, as the TC animals failed to control viremia and their viral loads increased. Although we had no direct measurements of viral diversity over the treatment period, we were able to use evolutionary analysis to infer PGD during this time. Crucially, and despite the low viral load seen in TC animals, we found that PGDs of TC animals not only tended to exceeded those of LC animals later in the study period, but also seemed to increase earlier. Although our results do not offer enough precision to definitively date the increase in PGD in every animal, it seems likely that this early increase in PGD in TC animals compared with LC animals occurs in the first 5 months.

This difference in evolutionary behaviour is repeated in the selected-sites analysis. TC macaques show a large number of positively-selected amino-acid replacements compared with LC animals; furthermore these substitutions occurred earlier than neutrally-selected substitutions. Mapping of the spatial location of the positively-selected codons to predicted gp120 structures indicated that these were located in solvent-exposed sites on the surface of the molecule, similar to data reported previously for HIV-1 gp120 [48], [49].

These differences in evolutionary dynamics between groups were also evident in the observed distributions of substitutions; V67M, and R751G appeared in all NC/TC macaques but none of the LC-derived viruses. In addition, mapping of these substitutions to the phylogenetic tree indicated that they had occurred early following infection. The R751G and V67M substitutions are associated with increased replicative capacity but the precise mechanisms remain to be determined [46].

Evolutionary theory predicts that population genetic diversity is directly proportional to population size only in the absence of selection. If this were true, then the greater diversity seen at later time points in TC and NC macaques would be a result of the increase in viremia in these animals, rather than its cause. However, at early time-points, our results strongly suggest that TC and NC viral populations have diversified whilst viral loads were low, a dynamic that can be explained by the generation of positively-selected amino-acid substitutions, which leads to greater population genetic variation and allows virus to escape immune control. Positive selection in viral populations is documented to occur in response to selection pressures by the host immune system and from treatment [50]. In this analysis; treatment, cytotoxic T cells and NAb activities may all have exerted selection pressures on the virus.

Within 2 months p.i. NAb active against SIVsm (strain SMM-3) were already detected in the NC/TC group of macaques. Neutralizing titers against SIVsm exceeded 1∶640 in all macaques by 4.5 months p.i. (2.5 months post-ART cessation) and reached levels as high as 1∶10240 in three macaques. Even though a heterologous NAb response against SIVmac251 and SIVdeltaB670 has previously been documented in SIVmac239 infected macaques [51], [52] and a response against SIVsm was expected due to its close phylogenetic relationship to SIVmac239 [45], neutralizing activity at such an early time point and of such high magnitude has not been reported before. However, the inoculate virus SIVmac239 could only be neutralized by IgG isolated after 5.5–7.5 months p.i. in the majority of animals. It therefore seems that prolonged exposure of the immune system to viral antigen is necessary in order to evoke NAb responses to SIVmac239.

The emergence of broadly cross-neutralizing antibodies in macaques at 6 to 8 months p.i. has previously been demonstrated [51], [52]. Our results support and extend these findings as we demonstrate the generation of broad NAb towards different SIV, HIV-2 and HIV-1 within 6 to 8 months. Antibody responses undergo complex maturation over time involving progressive changes in antibody avidity and conformational dependence [51], [52]. Antiretroviral treatment of macaques within this study has apparently impaired SIVmac239 replication capacity to levels corresponding to attenuated SIV strains used by Cole et al [52] resulting in a similarly long period (6–8 months) of affinity maturation. Hence, the findings presented here support the suggestion that time since infection and the presence of low to moderate viremia are factors contributing to the development of broadly reactive NAb [7], [8], [53].

Interestingly, the quality of NAb response, as reflected by breadth and potency, varied with the severity of SIV infection. Accordingly, viral control was associated with early development of broadly reactive NAb, whereas no or transient viral control was associated with higher potency. In addition, our findings provided insight as to the role of viral evolutionary factors contributing to the development of NAb activities. Analysis of sequence divergence from the inoculate strain revealed statistically significant differences between NC, TC and LC macaques. Viruses in plasma of NC3 accumulated a large number of amino acid mutations in *env*, reflected by a high level of divergence from SIVmac239. Divergence increased over time and also differed between all groups of macaques (NC, TC and LC) at 9 months p.i. NC/TC macaques showed greater levels of sequence divergence than LC macaques and the data suggest that PGD started to increase during the first 5 months in the NC/TC macaques. Therefore, we propose that the increase in viral divergence and diversity preceded the increase in viral load, and subsequently contributed to the strengthening and broadening of the NAb responses. Stratification of macaques into three distinct groups may bias outcomes when looking for associations. Therefore, macaques were ranked according to different functional parameters and viral characteristics and a multivariate analysis conducted. A Spearman's rank correlation determined that viral divergence in these macaques is strongly positively correlated with potency of the NAb responses. In macaques with more divergent viruses, the immune system has been exposed over time to a greater diversity of viral envelopes. Such diversity combined with appropriate antigen load may contribute to the overall magnitude NAb response.

Taken together, our results indicate that the macaques that controlled viral load displayed limited divergence, few positively-selected substitutions, slower CD4^+^ T-cell decline, and slower and less marked increase in PGD. Early on, they developed a broad NAb response although of limited potency, as compared to the transient controller group. Transient controller macaques, on the other hand, with higher viral loads later on, showed higher numbers of positively-selected sites, an early increase in PGD and greater divergence of SIV sequences. This correlated with higher potency NAb. Therefore, we suggest that the early increase in PGD preceded the increase in viral load after treatment withdrawal, subsequently setting the stage for development of potent NAb in these macaques. These results have implications for vaccine design and suggest that broad NAb but of low potency can be induced by relatively low antigen load with limited sequence diversity. Instead, the development of more potent NAb required higher diversity of antigens and included higher antigen load. These results also have implications for antiretroviral treatment and suggest that the increase in PGD started at an early stage of infection, either immediately following, or during, the treatment phase. We propose that this early phase of evolution is principally responsible for the later failure to control viremia.

## Methods

### Ethics statement

The animals were housed and handled at the Primate Research Centre of the Swedish Institute for Infectious Disease Control (Solna, Sweden) according to directives and guidelines of the Swedish Board of Agriculture, the Swedish Animal Protection Agency, The European Council Directive 86/609/EEC, and Convention ETS 123, including the revised Appendix A. The study was performed under approval of the Stockholm North Ethical Committee on Animal Experiments. The animals were housed in pairs in 4 m^3^ cages, enriched to give them the possibility to express their physiological and behavioural needs. They were habituated to the housing conditions for more than six weeks before the start of the experiment, and subjected to positive reinforcement training in order to reduce the stress associated with experimental procedures.

### Animals

Twelve four-year-old male Cynomolgus macaques (*Macaca fascicularis*) of Chinese origin were inoculated intravenously with 8000 MID50 of pathogenic cell-free SIVmac239 grown in rhesus macaque peripheral blood mononuclear cells (PBMCs) (kindly provided by Christiane Stahl-Hennig, Göttingen, Germany). The animals showed high viremia levels seven days post-inoculation (p.i.) and were subsequently treated from day 10 with a daily dose of 30 mg/kg of (R)-9 (2-phosphonylmethoxypropyl) adenine (PMPA) (tenofovir) given subcutaneously (Gilead Biosciences, CA, USA) for four weeks [54]. The tenofovir dose was thereafter reduced to 20 mg/kg and administered for 12 more weeks. The macaques were monitored for general clinical status, and blood samples were collected at four/six week intervals for analyses of viral load, CD4^+^ T-cell counts and NAb. The ExaVirLoad kit with a sensitivity of 1 fg reverse transcriptase (RT)/ml (400 copies/ml equivalents) of plasma was used for viral load determinations according to the manufacturer's instructions (Cavidi Tech AB, Uppsala, Sweden) [55]. CD4^+^ T-cell percentages and absolute CD4^+^ T-cell counts were analysed by flow cytometry using True Count tubes and CD45, CD4 and CD8 antibodies (BD Biosciences). Data was acquired on a FACSCalibur instrument using Cell Quest software (BD Biosciences).

### Viruses used in neutralization assays

SIVmac239 inoculate, SIVsm stock (sooty mangabey, strain SMM-3 originally obtained from P. Fultz and H. McClure, Yerkes National Primate Research Center, Atlanta, GA, USA [41], [42]), SIVmac251 [56] and two SIVsm re-isolates from a cynomolgus macaque, C39, that differed in their sensitivity to neutralization by autologous and heterologous sera from SIVsm-infected macaques were used [43]. SF162 (subtype B), 92BR025 (subtype C), 92UG024 (subtype D), CC030 and CC048 (CRF.02_AG) viruses were chosen as representatives of HIV-1 virus and the 1812 and 1682 viruses represent HIV-2 [57]. Virus stocks were prepared by infection of human PBMC activated with phytohemagglutinin for three days, as previously described [58]. Briefly, SIV-infected PBMC were cultured in RPMI 1640 medium (GIBCO Paisley, UK) with 10% fetal calf serum (FCS; Hyclone, Argentina), 10,000 IU/ml penicillin-streptomycin (Sigma, St. Louise, MO), 10 units/ml interleukin-2 (IL-2, Amersham Pharmacia Biotech, Sweden), and 2 µg/ml polybrene (Sigma, Germany). Cell free supernatants were collected seven and ten days after infection, aliquot and frozen at −80°C until use.

### Purification of total IgG from plasma

Plasma samples from SIVmac239-infected macaques were obtained from eleven time points: pre-inoculation, 1, 2, 3.5, 4.5, 5.5, 6.5, 7.5, 8.5, 10 and 14 months p.i. (Figure 1.A). Total immunoglobulin G (IgG) was used in all neutralization assays. IgG was purified by a method adapted from Dong *et al* [59]. In brief, 200 µl Protein G-Sepharose Fast Flow beads (GE Healthcare Bio-Sciences AB) were added to Eppendorf tubes and washed twice with 1 ml of sterile PBS. Plasma samples were first inactivated at 56°C for 30 minutes and then centrifuged at 4000×g for 20 min. 200 µl plasma and 200 µl PBS were added to the washed Protein G-Sepharose Fast Flow Beads and the mixture was incubated at room temperature for 1 h on a tube rotator. Thereafter plasma-bead mixtures were transferred to 0.45 µm-pore-size cellulose acetate filter Spin-X tubes (Costar) and centrifuged at 1000×g for 1 min, followed by one wash with 600 µl of PBS and finally centrifuged at 2000×g for 5 min to dry the beads. Neutralization buffer (20 µl, 1 M Tris-HCl, pH 9.0) was added to the bottom of fresh collection tubes and bound IgG was eluted with elution buffer (90 µl, 0.1 M glycine-HCl, pH 2.5) and centrifuged at 1000×g for 5 min into the neutralization buffer. Elution was repeated with another 90 µl of elution buffer and centrifuged at 2000×g for 5 min. Purified IgGs were kept at −20°C until use. Yield of IgG was analysed by ELISA [60]. Plates were coated overnight with AffiniPure goat anti-human IgG (20 µg/ml) (Jackson Immunotech). Alkaline phosphatase-conjugated anti-human IgG (diluted to 1∶5000) (Jackson Immunotech) was used as a detection antibody and macaque IgG (Rockland) was used as a standard. A positive control macaque serum (1YR) was a generous gift from Dr. Gerrit Koopman (BPRC, The Netherlands). Macaque 1YR had been infected by SIV-BK28 (a molecular clone of SIVmac251) five years earlier and was a long-term non-progressor [54]. The negative control was prepared by pooling plasma from three SIV-negative macaques.

### Neutralization assay in GHOST(3)-CCR5 cells

The GHOST(3)-CCR5 cell line has been derived from a human osteosarcoma cell (HOS) line by introducing the human CD4 gene and a chemokine receptor, here CCR5 [57]. The cells were also stably transfected with a vector construct encoding the green fluorescence protein (GFP) driven by the HIV-2_ROD_ LTR. Upon infection, the viral Tat protein activates the GFP marker and infected cells show green fluorescence. The GHOST(3)-CCR5 cell line was maintained in Dulbecco's modified Eagle's medium (DMEM; GIBCO) complemented with 7.5% FCS and 10,000 IU/ml penicillin-streptomycin in 25 cm^2^ culture flasks. The cultures were kept in a humidified atmosphere with 5% CO_2_ at 37°C. Monolayers were detached with 5 mM EDTA (pH 8.0) and split twice a week at a ratio of 1∶15–20. Cell lines were used for experiments within two months after thawing.

One day before infection the GHOST(3)-CCR5 cells were seeded into 96-well plates at a concentration of 5×10^3^ cells/well in 200 µl medium and incubated overnight at 37°C. Prior to infection, the medium was replaced with 50 µl fresh medium containing polybrene (2 µg/ml). Viruses were first titrated in five 5-fold dilution steps on the GHOST(3)-CCR5 cells to determine an appropriate virus concentration for the neutralization assays [43]. On the day of infection, virus was first diluted 5-fold in culture medium, followed by at least four 5-fold dilution steps, giving dilutions from 1/5 to 1/3125. Each dilution was added to triplicate wells at a volume of 150 µl per well and cultures were incubated overnight at 37°C. The day after infection, cultures were replaced with 200 µl fresh medium. Three days after infection, cultures were scored for individual numbers of cells or syncytia showing fluorescence (plaques) by fluorescence microscopy. Virus titers were calculated as plaque forming units (PFU) per ml: (average number of plaques in triplicate wells×virus dilution)/volume in the well [61]. For neutralization assays, IgG fractions of plasma and virus were mixed at dilutions that gave a final IgG concentration corresponding to IgG levels in diluted plasma and a virus concentration that gave between 10–90 fluorescent plaques per well. The virus and IgG mixtures were incubated at 37°C for one hour and subsequently titrated in triplicate (150 µl/well). The next day, plates were washed once and fresh medium added. At day three fluorescent plaques were counted under the fluorescent microscope. Neutralization was expressed as percentage of plaque reduction in the sample containing IgG, relative to virus without sample IgG and calculated using the formula: Plaque reduction (%) = [1−(PFU with sample IgG/PFU without sample IgG)]×100 [43]. Intra-assay variation has been assessed to establish the cut-off for neutralization to be used in the GHOST(3) assay. The standard deviation of the GHOST(3) assay range from 9.5% to 9.9% and applying >3SD as the cut-off results in 30% neutralization as the limit of detection [43], [61] and data not shown. The enhancement effect (Figure 3B) resulted in negative percentage neutralization values; logistic regression analyses indicated that the optimal fitting of the neutralization curves gives a median neutralization value of approximately 30%.

### Potency and breadth analysis

For analysis of the magnitude of NAb responses, IgG samples were titrated and used in neutralization assays against 4 SIV (SIVmac239 inoculate, SIVsm stock, SIVsm:C39^sens^ and SIVsm:C39^res^), two HIV-2 (1682 and 1812) as well as 5 HIV-1 (SF162, 92BR025, 92UG024, CC030 and CC048) viruses. The magnitude of an individual IgG sample was determined by its neutralization score defined as log-transformed titers [9]. Log-transformed titers were calculated by dividing the highest neutralizing titer values by 100 before applying a log-base 3 transformation and then adding 1 [Y = log3 (dilution/100)+1]. All titers below the limit of detection were given a value of 33 to calculate a neutralization score. The potency score for each macaque was calculated by summarizing the magnitude (neutralization score) against each virus and then dividing by total number of viruses tested in Table 2 (n = 8). Breadth of an individual IgG sample was defined by the number of viruses neutralized at a 1∶20 dilution.

### Isolation and PCR amplification of *env* from viral RNA

RNA was isolated from plasma samples using the QIAamp Viral RNA Mini Kit (Qiagen) as per the manufacturer's protocol. 140 µl plasma or supernatant was used for isolation and RNA was eluted in a final volume of 40 µl. Viral RNA was amplified in a one-step RT-PCR reaction using Superscript III One-step RT-PCR with High Fidelity Platinum *Taq* (Invitrogen). Reactions consisted of an RT step of 55°C for one hour, denaturation of two minutes at 94°C, followed by 40 cycles of PCR of the whole *env* gene with SIVF (5′-CTG CAT CAA ACA AGT AAG TAT GGG ATG TCT TGG G-3′) and SIVR (5′-CAT ATA CTG TCC CTG ATT GTA TTT CTG TCC CTC-3′). When samples gave no visible band following electrophoresis, a first round of RT-PCR was carried out using external primers (SIVextF: 5′-ATC CTC TCT CAG CTA TAC CGC C-3′ and SIVextR: 5′-GAT GAG TAA GAT GAT GAC TTG GA GGG-3′), followed by a second round of PCR with SIVF and SIVR using Advantage 2 Polymerase mix (Clontech). In order to ensure that all DNA strands were freshly generated, and hence homoduplexes, purified PCR product underwent an additional round of PCR with SIVF and SIVR primers containing an additional CAT ‘identifier tag’ sequence at the 5′ end. PCR product (2.7kb) was gel purified using the QIAquick Gel Extraction Kit (Qiagen) according to the manufacturer's protocol.

### Cloning and sequencing of *env* PCR products

Purified PCR product was cloned into the pCR®4-TOPO vector from the TOPO TA Cloning® Kit for Sequencing (Invitrogen) according to the manufacturer's protocol. Following transformation into One-shot TOP10 chemically competent *E.coli*, colonies were selected on ampicillin. Colonies were picked and shipped to Functional Biosciences, Inc, Madison, WI, USA for sequencing. Plasmid DNA was extracted and sequenced using the four primers (M13F (5′-GTA AAA CGA CGG CCA G -3′) M13R (5′- CAG GAA ACA GCT ATG AC-3′) SIV_seqF (5′-TGT CAT ATT AGA CAA ATA ATC AAC AC-3′) and SIV_seqR (5′-AAT CGA TAC AGT TCT GCC ACC TCT GC-3′).

### Sequence assembly

The data was assembled into full-length sequences (contigs) using a custom scripts that automatically ran pregap4 (http://staden.sourceforge.net/manual/pregap4_unix_toc.html), extracted open reading frames (ORFS) from the contigs using the EMBOSS tool getorf [48] and aligned the ORFS (DNA and protein) using MUSCLE [62]. This approach generated a single sequence for each clone, spanning the entire *env* gene. Incomplete sequences were discarded. Sequence data are available from GenBank under accession numbers HM800143 to HM800423.

### CD4^+^ correlation with viral load

The non-parametric Spearman correlation test was used to test for correlation between CD4^+^ T-cell population and viral load. The different groups of macaques were compared by using the Mann-Whitney non-parametric test using SPSS statistical software. In addition, the data obtained over time were analyzed using the two-way repeated measures analysis of variance model.

### Initial phylogenetic analyses

We used phylogenetic analyses to infer viral evolutionary history in *env*, particularly, to distinguish amino acid replacements under positive selection from those occurring at random. Our initial phylogenetic analysis checked the quality of the input data, and then sought to obtain a robust estimate of the best phylogeny, substitution model and substitution model parameters as a solid foundation for the later selection and epistasis analyses. During the phylogeny reconstruction the assembled sequences were aligned in MUSCLE [62] and inspected visually in Se-Al (A. Rambaut: http://tree.bio.ed.ac.uk/software/seal). First-pass phylogenetic reconstruction was carried out in PHYML [63] under the HKY+γ model with empirical base frequencies. This phylogeny was inspected in FigTree (http://tree.bio.ed.ac.uk/software/figtree) and all sequences with excessively long branches removed from the analysis. ModelTest [64]–[66] (implemented in HYPHY [67] was used to identify the best-fitting model for the data, by Akaike information criterion (AIC) and hierarchical analyses. This model (GTR+γ+I) was used in all future analyses wherever possible. The phylogeny and model parameters for this refined data set were then iteratively optimised in GARLI [68] http://www.bio.utexas.edu/faculty/antisense/garli/Garli.html) and PHYML, respectively. Alignment diversity was scored for each site in the nucleotide and codon alignments using the Shannon entropy index, implemented in the program Shannon ([69] J.Parker http://evolve.zoo.ox.ac.uk/evolve/SHiAT.html). Pair-wise divergence between sequences and the root (SIVmac239) sequence was calculated in HYPHY under the GTR+γ+I model.

### Detection of selection

The direction (positive/neutral/negative) and strength of selection can be inferred from the ratio of the rate of nucleotide mutations leading to non-synonymous (dN) or synonymous (dS) amino-acid changes (substitutions). In general, at sites where dN is much greater than dS positive selection is likely to have occurred. Conversely, at sites where dN is much less than dS conservation (negative selection) is inferred. Global (*env*) and site-wise dN/dS ratios were estimated from the best available topologies in HYPHY. The best-supported nucleotide substitution model identified above (GTR+γ+I) was fitted to the data to estimate the nucleotide states at ancestral nodes in the phylogeny. Next, site-wise dN/dS ratios and significances were estimated by single likelihood ancestor counting (SLAC) in HYPHY with ambiguities in ancestral sequences resolved by averaging and using a single-parent node structure [67].

### Arrival time of positively-selected amino-acid substitutions

For each animal the earliest time following infection at which each amino-acid substitution occurred (‘arrival time’) was reconstructed in following way: we matched the ancestral sequence inferred at each internal node in the phylogeny with its mean posterior estimated date of existence; for each site in the amino-acid alignment, we then calculated the earliest time at which any amino-acid substitution away from the parental (SIVmac239) sequence occurred within each macaque's sub-tree. Subsequent wild-type reversions were ignored; we grouped amino-acid substitutions' arrival times according to whether they occurred at sites predicted to be subject to positive selection or simply represented neutral evolution. As a visual reference, we also mapped the first occurrence of positively-selected substitutions in the sub-tree representing each macaque onto the phylogeny (Figure S4).

### Estimating population genetic diversity

Our samples were drawn at three time points (2, 5.5 and 9 months p.i. as well as the parental virus), and consequently do not evenly cover the entire study period resulting in large confidence intervals. Therefore, we did not examine temporal fluctuations in viral diversity directly. Fortunately, Bayesian Markov-chain Monte Carlo (MCMC) techniques employing the coalescent [70] are able to exploit temporally-spaced sequence data to estimate ancestral population genetic diversity (PGD) at all points in time, not just those time points at which samples were taken. Therefore we have also analysed the data under a coalescent Bayesian MCMC analysis in BEAST v1.4.8 [71] using a relaxed molecular clock model (a model of evolution that in contrast to the strict clock model (a single substitution rate applied across the phylogeny) allows substitution rates to vary across the phylogeny) [72] and constraining the tree so that individual macaques' sequences formed exclusive sub-trees (as monophyletic groups). Having estimated substitution model and mean evolutionary rate parameters jointly from this data-set, we then repeated the BEAST analysis separately for individual animals, fixing these substitution model and rate parameters for individual animals, this time using the Bayesian skyline plot reconstruction to estimate ancestral population sizes. All BEAST MCMC chains were constructed from 30×10^6^ states to give 30,000 samples, with 10% discarded for burn-in (to ensure the MCMC sampler had begun to sample from the true posterior parameter distributions correctly). Several chains were run separately to check convergence; traces were inspected by hand to verify that a 10% burn-in was sufficient and combined. We estimated the *env* sequence diversity and absolute size of the viral population in each animal during the treatment phase and obtained the sum of PGD estimates (time-integral of PGD) during the treatment phase.

### Structural patterns of SIV evolution

A molecular model of the monomeric SIV gp120 was generated using the crystal structure of the HIV-1 gp120 (PDB ID 2B4C) and the SWISS-MODEL protein modeling server [73]. The program Coot was further used for small localized structural refinements [74]. The trimeric model of SIV gp120 was created using a previously published model of the HIV-1 gp120 trimer kindly provided by Dr Peter D. Kwong and Dr Marie Pancera. Positively- and negatively-selected sites were mapped on the gp120 models and potential N-linked glycosylation sites were identified using the N-GlycoSite prediction tool from the HIV sequence database (http://www.hiv.lanl.gov/content/sequence/GLYCOSITE/glycosite.html) [75]. Figures were created using Pymol (DeLano Scientific LLC, Palo Alto, USA).

### Positive selection in hypervariable (V) regions

We investigated the potential disproportionate clustering of positively-selected substitutions in the hypervariable (V) regions of the *env* gene. In order to retain statistical power while allowing for false positives arising from the large number of hypothesis tests these data represent, we opted to compare the empirical distribution of *p*-values in support of positive selection between these regions. We calculated empirical cumulative distribution functions (eCDF) on the confidence measures (*p*-values) for positive selection in individual amino acids, both for the whole alignment and for subsets covering each of the five recognised V regions. The eCDF function orders the observed *p*-values and then scores the cumulative proportion of total observed values that fall into discrete bins: a series of ranges from 0–1 in small (0.01) increments (0–0.01, 0.01–0.02, to 1). Larger functions at low *p*-values indicate greater significance. The eCDFs were compared to the eCDF for *p*-values in the whole *env* gene using the one-sided Kolmogorov-Smirnov distribution difference test (H_a_: D_all_>D_V_).

### Correlation between evolutionary and clinical factors (multivariate analysis)

Animals were assigned ranks based on each of the clinical (viral load at 6.5 months; CD4^+^ T- cell decline up to 6.5 months and CD4 counts at 6.5 months; NAb breadth and potency) and evolutionary (diversity; divergence; number of positively-selected mutations; time-integral of PGD) indicators. A Spearman rank-correlation was used to determine if there were any relationships between evolutionary and clinical factors. To control for false-positives, we employed the multiple test correction procedure used by Benjamini and Hochberg [44].

## Supporting Information

Figure S1 CD4^+^ T cells in macaques after SIVmac239 infection. Kinetic analyses of peripheral blood CD4^+^ T cells during SIVmac239 infection. CD4 counts are depicted longitudinally for each animal (LC; green, TC; blue and NC; red). Tenofovir treatment period is indicated by shaded area.
(0.41 MB TIF)

Figure S2 Heterologous neutralization of HIV-2. Purified IgG obtained from plasma samples 14 months post-inoculation were titrated and analyzed for neutralization of HIV-2 1812 (A) and HIV-2 1682 (B). Neutralization profiles of NC (animal 3, red) and TC (animals 1, 5, 8, 11 blue) as well as LC (animals 2, 4, 6, 7, 9, 10, 12, green) are shown. Values are means of two independent assays. Assay cut-off was 30% as indicated by line.
(0.40 MB TIF)

Figure S3 Sequence positions of mutations in re-isolates from NC3. *Env* sequences from virus re-isolated from macaques 3 at 4.5 months p.i. were compared with that of SIVmac239 in order to identify escape mutations that could account for the observed changes in neutralization sensitivity. NC3 sequences displayed a number of sequence changes including V67M, A417T and R751G. Sequences obtained from NC3 at 4.5 months (neutralization resistant) and 9 months (neutralization sensitive) were also compared. Escape mutations that confer resistance were still present suggesting a broadening of the NAb response at the later time point.
(0.39 MB TIF)

Figure S4 Phylogeny reconstruction and arrival times of significant positively-selected codon substitutions. Phylogeny reconstruction: Maximum clade credibility (MCC) tree of 281 SIV *env* sequences (12 hosts) plus inoculate (SIVmac239). MCC tree resolved from posterior set of 9000 trees (PST) sampled from the posterior distribution in BEAST. Sequences from each host constrained to be monophyletic. Model parameters: Substitution - HKY85+gamma (4 rate categories); demographic - exponential growth; molecular clock type - uncorrelated lognormal distribution (UCLN; ‘relaxed’ clock); branch lengths in average nucleotide substitutions. Sub-trees corresponding to individual macaques are shown in various colours. Arrival times: Starred (‘*’) nodes represent the earliest estimated arrival time significantly positively-selected codon substitution (neutrally-selected substitutions and reversions not shown; see Methods).
(6.82 MB TIF)

Figure S5 Empirical cumulative density functions (eCDF) of *p*-values for positively-selected amino-acid substitutions. The eCDF is a binning function that scores the proportion of total data points (vertical axis) of equal or lesser *p* with increasing values of *p* from 0 to 1 (horizontal axis). Low-valued (significant *p*) data sets are expected to plateau early, while high-valued (not significant *p*) data sets will plateau late. Black: all *env* sites; red V1; blue V2; green V3; brown V4, and grey V5.
(0.14 MB TIF)

Table S1 Number of *env* sequences obtained from plasma samples and re-isolated virus for macaques 1–12.
(0.04 MB DOC)

Table S2 Positive and negative selection in SIV *env*.
(0.15 MB DOC)

Table S3 Epistasis in SIV *env*.
(0.09 MB DOC)
