# Supplementary figures and images for: Generation of Neutralizing Antibodies and Divergence of SIVmac239 in Cynomolgus Macaques Following Short-Term Early Antiretroviral Therapy
Source: PLoS Pathog. 2010 Sep 2;6(9):e1001084. doi: 10.1371/journal.ppat.1001084 (PMC2932721; doi:10.1371/journal.ppat.1001084)

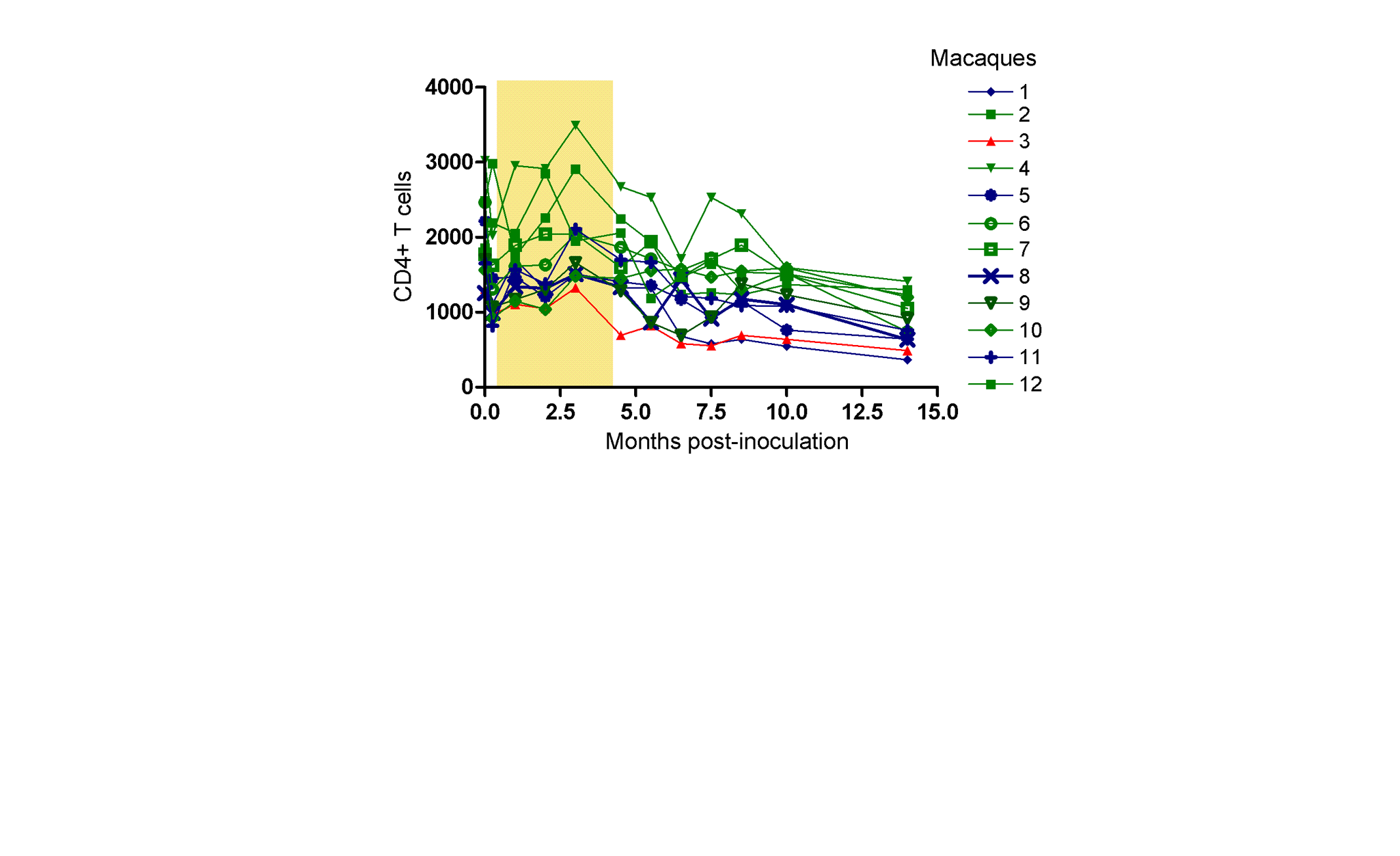

Supplement: Figure S1 — CD4+ T cells in macaques after SIVmac239 infection. Kinetic analyses of peripheral blood CD4+ T cells during SIVmac239 infection. CD4 counts are depicted longitudinally for each animal (LC; green, TC; blue and NC; red). Tenofovir treatment period is indicated by shaded area. (0.41 MB TIF) [file ppat.1001084.s001.tif]

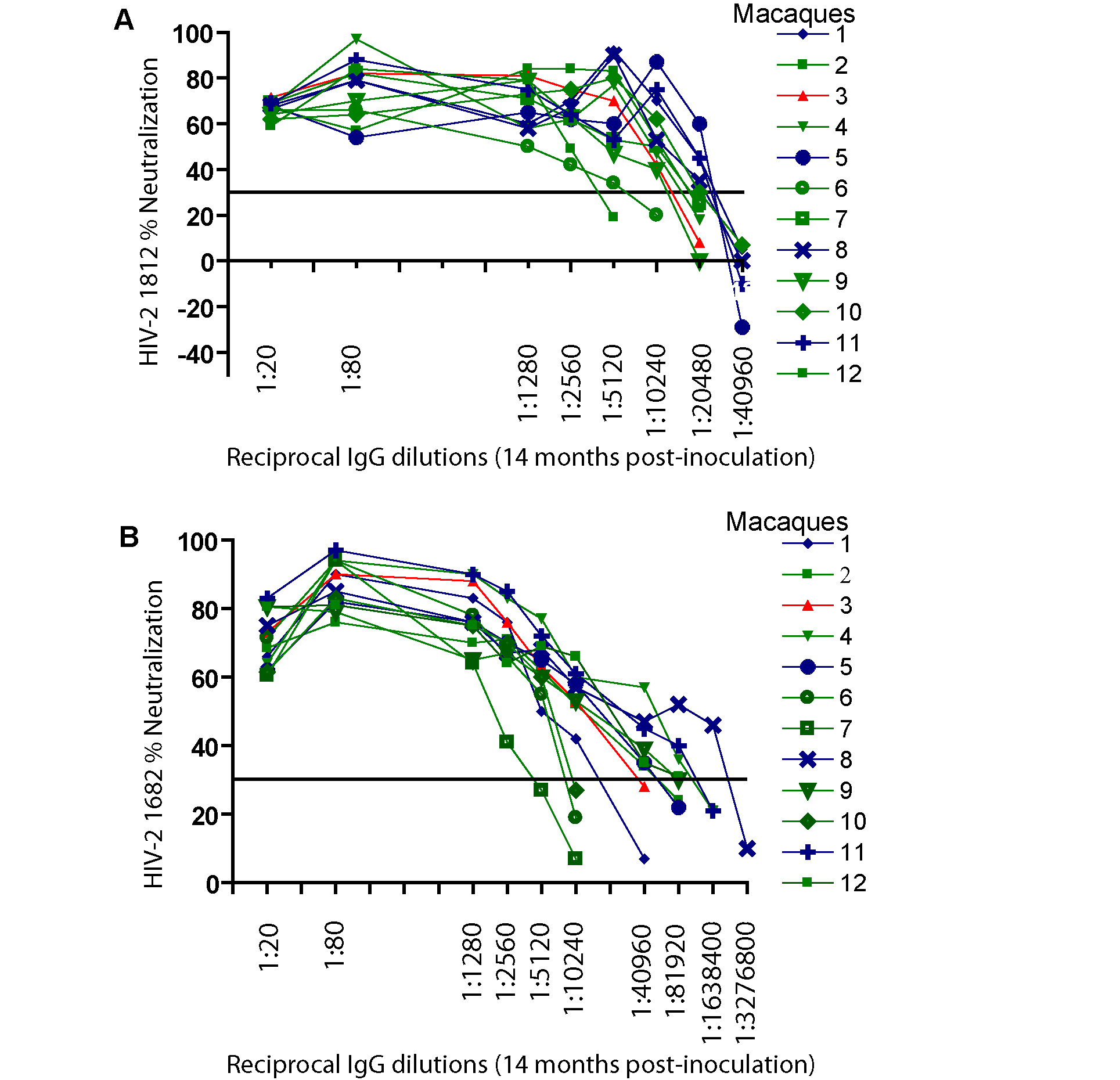

Supplement: Figure S2 — Heterologous neutralization of HIV-2. Purified IgG obtained from plasma samples 14 months post-inoculation were titrated and analyzed for neutralization of HIV-2 1812 (A) and HIV-2 1682 (B). Neutralization profiles of NC (animal 3, red) and TC (animals 1, 5, 8, 11 blue) as well as LC (animals 2, 4, 6, 7, 9, 10, 12, green) are shown. Values are means of two independent assays. Assay cut-off was 30% as indicated by line. (0.40 MB TIF) [file ppat.1001084.s002.tif]

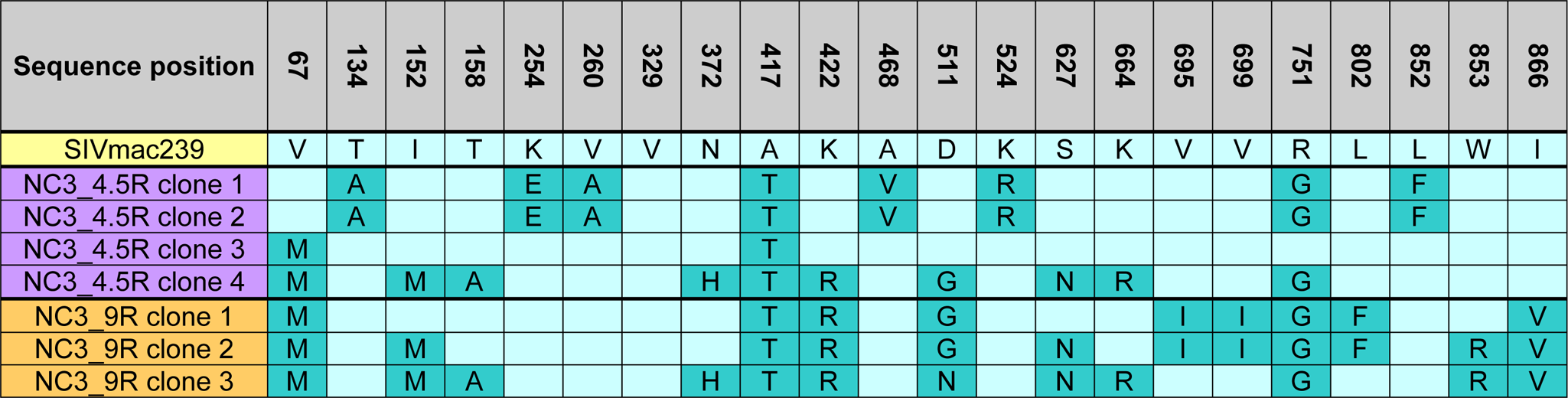

Supplement: Figure S3 — Sequence positions of mutations in re-isolates from NC3. Env sequences from virus re-isolated from macaques 3 at 4.5 months p.i. were compared with that of SIVmac239 in order to identify escape mutations that could account for the observed changes in neutralization sensitivity. NC3 sequences displayed a number of sequence changes including V67M, A417T and R751G. Sequences obtained from NC3 at 4.5 months (neutralization resistant) and 9 months (neutralization sensitive) were also compared. Escape mutations that confer resistance were still present suggesting a broadening of the NAb response at the later time point. (0.39 MB TIF) [file ppat.1001084.s003.tif]

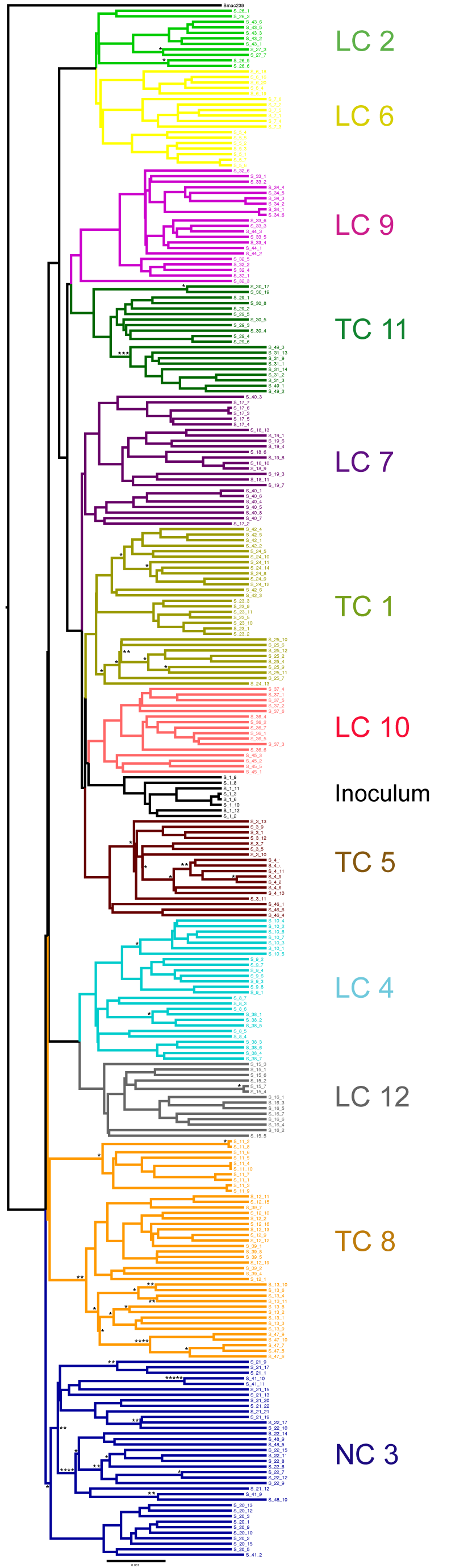

Supplement: Figure S4 — Phylogeny reconstruction and arrival times of significant positively-selected codon substitutions. Phylogeny reconstruction: Maximum clade credibility (MCC) tree of 281 SIV env sequences (12 hosts) plus inoculate (SIVmac239). MCC tree resolved from posterior set of 9000 trees (PST) sampled from the posterior distribution in BEAST. Sequences from each host constrained to be monophyletic. Model parameters: Substitution - HKY85+gamma (4 rate categories); demographic - exponential growth; molecular clock type - uncorrelated lognormal distribution (UCLN; ‘relaxed’ clock); branch lengths in average nucleotide substitutions. Sub-trees corresponding to individual macaques are shown in various colours. Arrival times: Starred (‘*’) nodes represent the earliest estimated arrival time significantly positively-selected codon substitution (neutrally-selected substitutions and reversions not shown; see Methods). (6.82 MB TIF) [file ppat.1001084.s004.tif]

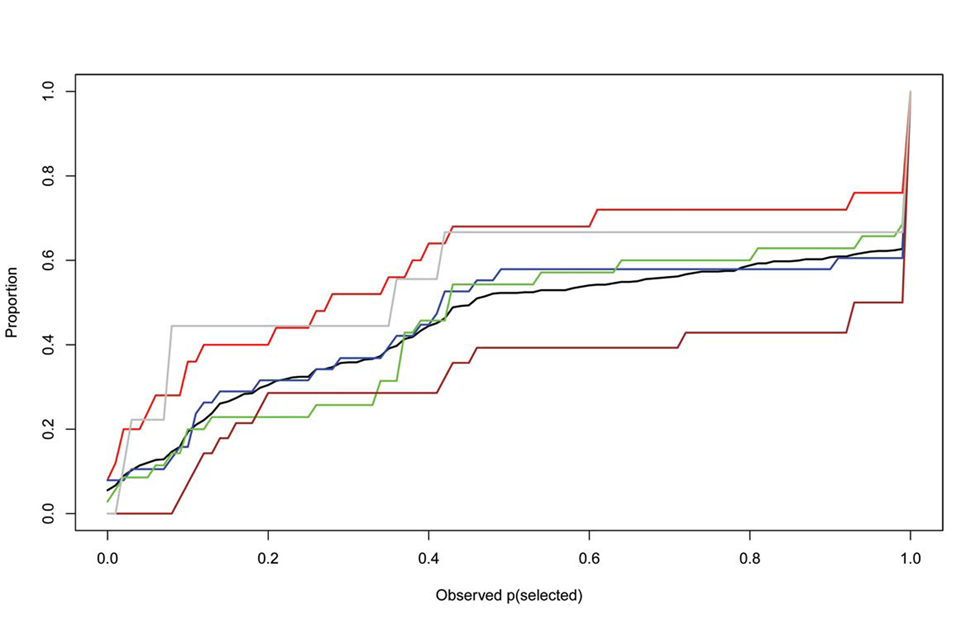

Supplement: Figure S5 — Empirical cumulative density functions (eCDF) of p-values for positively-selected amino-acid substitutions. The eCDF is a binning function that scores the proportion of total data points (vertical axis) of equal or lesser p with increasing values of p from 0 to 1 (horizontal axis). Low-valued (significant p) data sets are expected to plateau early, while high-valued (not significant p) data sets will plateau late. Black: all env sites; red V1; blue V2; green V3; brown V4, and grey V5. (0.14 MB TIF) [file ppat.1001084.s005.tif]
