# Supplementary material for: Generation of Neutralizing Antibodies and Divergence of SIVmac239 in Cynomolgus Macaques Following Short-Term Early Antiretroviral Therapy
Source: PLoS Pathog. 2010 Sep 2;6(9):e1001084. doi: 10.1371/journal.ppat.1001084 (PMC2932721; doi:10.1371/journal.ppat.1001084)
Supplement: Table S1 — Number of env sequences obtained from plasma samples and re-isolated virus for macaques 1–12. (0.04 MB DOC) [file ppat.1001084.s006.doc]

| **Macaque** | **Number of full-length *env* sequences** | | | | |
| --- | --- | --- | --- | --- | --- |
|  | **2 months** | **5.5 months** | **9 months** | **4.5 month re-isolate** | **9 month re-isolate** |
| **1** | 7 | 8 | 8 | 6 | no isolate |
| **2** | 4 | 2 | 0 | 5 | no isolate |
| **3** | 9 | 10 | 10 | 4 | 3 |
| **4** | 5 | 7 | 7 | 7 | no isolate |
| **5** | 0 | 8 | 7 | no isolate | 3 |
| **6** | 7 | 5 | 6 | no isolate | no isolate |
| **7** | 6 | 5 | 6 | 7 | no isolate |
| **8** | 10 | 10 | 9 | 6* | 5 |
| **9** | 6 | 6 | 6 | 3 | no isolate |
| **10** | 0 | 6 | 6 | 4 | 0 |
| **11** | 6 | 5 | 6 | no isolate | 3 |
| **12** | 0 | 7 | 7 | no isolate | no isolate |

**Table S1**. **Number of *env* sequences obtained from plasma samples and re-isolated virus for macaques 1-12.**

*re-isolated virus was not infectious
