# Supplementary material for: Generation of Neutralizing Antibodies and Divergence of SIVmac239 in Cynomolgus Macaques Following Short-Term Early Antiretroviral Therapy
Source: PLoS Pathog. 2010 Sep 2;6(9):e1001084. doi: 10.1371/journal.ppat.1001084 (PMC2932721; doi:10.1371/journal.ppat.1001084)
Supplement: Table S2 — Positive and negative selection in SIV env. (0.15 MB DOC) [file ppat.1001084.s007.doc]

**Table S2**. **Positive and negative selection in SIV *env.***

**A) Significantly positively-selected (‘diversifying’ selection; dN >> dS) codons in the data set.**

| **codon** | **SIVmac239** | **dN** | **dS** | **dN/dS** | **P(pos, FDR)** |
| --- | --- | --- | --- | --- | --- |
| 7 | Q | 4.1899 | 0 | inf | 0.0339 |
| 36 | R | 4.0815 | 0 | inf | 0.0187 |
| 67 | V | 36.1266 | 7.56403 | 4.77611 | 0.0001 |
| 116 | S | 6.4111 | 0 | inf | 0.0166 |
| 134 | T | 9.0015 | 1.50366 | 5.98641 | 0.0263 |
| 136 | T | 8.9047 | 1.18226 | 7.53194 | 0.0097 |
| 190 | A | 3.9216 | 0 | inf | 0.0353 |
| 254 | K | 20.4236 | 0 | inf | 0.0000 |
| 260 | V | 24.3611 | 0 | inf | 0.0000 |
| 310 | K | 6.4842 | 0 | inf | 0.0109 |
| 338 | R | 4.0916 | 0 | inf | 0.0186 |
| 341 | Q | 8.6846 | 0 | inf | 0.0009 |
| 371 | N | 4.2235 | 0 | inf | 0.0229 |
| 372 | N | 10.7931 | 0 | inf | 0.0023 |
| 374 | D | 5.4791 | 0 | inf | 0.0273 |
| 375 | K | 4.3201 | 0 | inf | 0.0388 |
| 417 | A | 22.3846 | 1.74866 | 12.801 | 0.0001 |
| 420 | K | 4.4032 | 0 | inf | 0.0191 |
| 421 | P | 11.3854 | 1.50934 | 7.54326 | 0.0092 |
| 422 | K | 9.4493 | 0 | inf | 0.0006 |
| 458 | T | 3.1327 | 0 | inf | 0.0381 |
| 468 | A | 3.8931 | 0 | inf | 0.0359 |
| 474 | D | 10.4159 | 0 | inf | 0.0026 |
| 476 | N | 7.8435 | 0 | inf | 0.0020 |
| 502 | I | 10.2937 | 0 | inf | 0.0003 |
| 511 | D | 14.4903 | 4.02717 | 3.59812 | 0.0411 |
| 524 | K | 21.2143 | 0 | inf | 0.0000 |
| 528 | F | 17.3954 | 0 | inf | 0.0001 |
| 550 | L | 2.6224 | 0 | inf | 0.0409 |
| 650 | N | 4.8941 | 0 | inf | 0.0385 |
| 751 | R | 7.4797 | 0 | inf | 0.0057 |
| 776 | L | 6.6647 | 0.7116 | 9.36584 | 0.0147 |
| 779 | L | 3.8749 | 0 | inf | 0.0257 |
| 780 | L | 15.7515 | 0.804504 | 19.5792 | 0.0000 |
| 802 | L | 11.9023 | 0 | inf | 0.0001 |
| 807 | A | 6.1760 | 0 | inf | 0.0031 |
| 835 | Q | 5.5998 | 0 | inf | 0.0138 |
| 849 | W | 6.1633 | 0 | inf | 0.0032 |
| 866 | I | 7.4093 | 0 | inf | 0.0021 |

**B) Significantly negatively-selected (‘purifying’, or ‘conserving’ selection; dN << dS) sites in the data set.**

| **codon** | **SIVmac239** | **dN** | **dS** | **dN/dS** | **P(neg, FDR)** |
| --- | --- | --- | --- | --- | --- |
| 8 | L | 0 | 6.5082 | 0 | 0.0050 |
| 40 | I | 0 | 3.6473 | 0 | 0.0215 |
| 44 | C | 0 | 3.9247 | 0 | 0.0208 |
| 45 | A | 0 | 4.5847 | 0 | 0.0048 |
| 81 | T | 0 | 4.5573 | 0 | 0.0066 |
| 100 | P | 0.70813 | 7.0158 | 0.100934 | 0.0180 |
| 103 | K | 0 | 3.3153 | 0 | 0.0280 |
| 139 | S | 0 | 4.5693 | 0 | 0.0117 |
| 141 | K | 0 | 3.3193 | 0 | 0.0280 |
| 142 | V | 0 | 10.8801 | 0 | 0.0000 |
| 169 | K | 0 | 4.9996 | 0 | 0.0071 |
| 181 | K | 0.704952 | 8.5095 | 0.082843 | 0.0063 |
| 189 | S | 0 | 10.6778 | 0 | 0.0002 |
| 197 | G | 0 | 23.4384 | 0 | 0.0000 |
| 249 | S | 0.804747 | 9.3255 | 0.086295 | 0.0049 |
| 255 | C | 0.697123 | 5.9449 | 0.117264 | 0.0417 |
| 259 | V | 0 | 2.3010 | 0 | 0.0371 |
| 271 | T | 0 | 3.4616 | 0 | 0.0214 |
| 314 | P | 0 | 4.5497 | 0 | 0.0089 |
| 315 | G | 0 | 3.0216 | 0 | 0.0202 |
| 355 | K | 0 | 5.0352 | 0 | 0.0070 |
| 363 | K | 2.13311 | 10.1971 | 0.209187 | 0.0228 |
| 376 | I | 0.660143 | 5.0455 | 0.130837 | 0.0369 |
| 377 | N | 2.07908 | 20.6417 | 0.100722 | 0.0001 |
| 427 | R | 0 | 6.1157 | 0 | 0.0008 |
| 432 | C | 0 | 3.9120 | 0 | 0.0209 |
| 442 | H | 0 | 3.9193 | 0 | 0.0258 |
| 455 | G | 0 | 3.0290 | 0 | 0.0202 |
| 460 | N | 0 | 2.6167 | 0 | 0.0393 |
| 492 | L | 0 | 4.0544 | 0 | 0.0268 |
| 494 | L | 1.27781 | 9.9593 | 0.128304 | 0.0095 |
| 534 | G | 0.520703 | 14.2903 | 0.036438 | 0.0000 |
| 556 | T | 0 | 10.5909 | 0 | 0.0001 |
| 598 | K | 0 | 12.8011 | 0 | 0.0000 |
| 613 | F | 0 | 5.9106 | 0 | 0.0063 |
| 620 | T | 0 | 27.6485 | 0 | 0.0000 |
| 657 | E | 0 | 7.5743 | 0 | 0.0003 |
| 671 | K | 0 | 3.7222 | 0 | 0.0137 |
| 707 | V | 0 | 2.3006 | 0 | 0.0371 |
| 753 | G | 0.520943 | 40.8197 | 0.012762 | 0.0000 |
| 756 | G | 0 | 2.4109 | 0 | 0.0287 |
| 757 | G | 0.520805 | 5.2404 | 0.099383 | 0.0282 |
| 803 | Q | 0 | 4.9957 | 0 | 0.0238 |
| 814 | E | 0 | 11.9951 | 0 | 0.0000 |
| 856 | L | 0 | 3.4585 | 0 | 0.0301 |
| 860 | G | 2.65783 | 14.1890 | 0.187317 | 0.0024 |
| 869 | R | 0 | 4.8609 | 0 | 0.0026 |
| 871 | R | 0 | 3.2146 | 0 | 0.0216 |
| 876 | L | 0.7691 | 16.3557 | 0.047023 | 0.0000 |

The total set of significant (*p* ≤ 0.05) values for positively or negatively-selected sites was filtered by the false discovery rate (FDR) correction method described in the Methods; only those sites remaining following FDR correction are shown. Full results are available on request.
