# Supplementary material for: Generation of Neutralizing Antibodies and Divergence of SIVmac239 in Cynomolgus Macaques Following Short-Term Early Antiretroviral Therapy
Source: PLoS Pathog. 2010 Sep 2;6(9):e1001084. doi: 10.1371/journal.ppat.1001084 (PMC2932721; doi:10.1371/journal.ppat.1001084)
Supplement: Table S3 — Epistasis in SIV env. (0.09 MB DOC) [file ppat.1001084.s008.doc]

| **Site1** | **Site2** | **Total posterior epistasis probability** |  | **Site1** | **Site2** | **Total posterior epistasis probability** |
| --- | --- | --- | --- | --- | --- | --- |
| 623 | 662 | 0.996137 |  | 480 | 820 | 0.62837 |
| 197 | 603 | 0.983848 |  | 10 | 28 | 0.626834 |
| 536 | 685 | 0.941087 |  | 292 | 316 | 0.615663 |
| 364 | 497 | 0.940763 |  | 33 | 527 | 0.614419 |
| 332 | 633 | 0.905279 |  | 622 | 635 | 0.609269 |
| 197 | 319 | 0.826716 |  | 145 | 723 | 0.601417 |
| 460 | 497 | 0.823556 |  | 745 | 760 | 0.577903 |
| 248 | 455 | 0.813183 |  | 205 | 315 | 0.566254 |
| 254 | 510 | 0.80018 |  | 159 | 620 | 0.564813 |
| 149 | 603 | 0.780321 |  | 150 | 710 | 0.558263 |
| 460 | 824 | 0.757721 |  | 239 | 522 | 0.552248 |
| 111 | 757 | 0.744027 |  | 261 | 735 | 0.550286 |
| 131 | 510 | 0.741909 |  | 27 | 693 | 0.549323 |
| 84 | 684 | 0.74072 |  | 370 | 648 | 0.548648 |
| 719 | 737 | 0.738293 |  | 27 | 251 | 0.546899 |
| 106 | 402 | 0.734225 |  | 122 | 664 | 0.542029 |
| 208 | 828 | 0.730537 |  | 86 | 664 | 0.538569 |
| 320 | 544 | 0.730419 |  | 18 | 735 | 0.537813 |
| 263 | 713 | 0.724837 |  | 86 | 122 | 0.536248 |
| 472 | 521 | 0.724582 |  | 270 | 704 | 0.530807 |
| 154 | 526 | 0.720675 |  | 126 | 710 | 0.530256 |
| 566 | 830 | 0.704365 |  | 538 | 648 | 0.527775 |
| 511 | 568 | 0.69859 |  | 126 | 150 | 0.527272 |
| 54 | 109 | 0.696869 |  | 690 | 745 | 0.526629 |
| 329 | 769 | 0.687992 |  | 214 | 517 | 0.525412 |
| 676 | 834 | 0.680978 |  | 116 | 270 | 0.524015 |
| 303 | 382 | 0.68077 |  | 197 | 365 | 0.522972 |
| 498 | 716 | 0.679825 |  | 85 | 457 | 0.522746 |
| 199 | 394 | 0.673362 |  | 159 | 486 | 0.522716 |
| 394 | 432 | 0.673051 |  | 486 | 620 | 0.521648 |
| 576 | 795 | 0.671977 |  | 251 | 693 | 0.519831 |
| 456 | 525 | 0.671856 |  | 18 | 261 | 0.517895 |
| 107 | 506 | 0.664112 |  | 690 | 760 | 0.516161 |
| 196 | 767 | 0.663587 |  | 249 | 395 | 0.515165 |
| 533 | 781 | 0.655238 |  | 550 | 749 | 0.512294 |
| 199 | 432 | 0.648176 |  | 337 | 387 | 0.509803 |
| 13 | 765 | 0.645827 |  | 423 | 586 | 0.509669 |
| 226 | 513 | 0.638771 |  | 133 | 411 | 0.505012 |
| 194 | 460 | 0.631555 |  | 342 | 720 | 0.501386 |

**Table S3. Epistasis in SIV *env***

The epistatic interactions with significant total posterior support (HYPHY BGM co-evolution method). Codon positions are given relative to 5’ AUG in SIVmac239 *env.* The total set of significant (*p* ≤ 0.05) values for sites under epistasis was filtered by the false discovery rate (FDR) correction method described in the Methods; only those sites remaining following FDR correction are shown. Full results are available on request.
